# Is there an association between hospital staffing levels and inpatient-COVID-19 mortality rates?

**DOI:** 10.1371/journal.pone.0275500

**Published:** 2022-10-19

**Authors:** Mona Al-Amin, Md. Nazmul Islam, Kate Li, Natalie Shiels, John Buresh

**Affiliations:** 1 Healthcare Administration, Suffolk University, Boston, Massachusetts, United States of America; 2 Optum Labs, UnitedHealth Group, Minnetonka, Minnesota, United States of America; 3 Information Systems and Operations Management, Sawyer Business School, Suffolk University, Boston, Massachusetts, United States of America; Stanford University School of Medicine, UNITED STATES

## Abstract

**Objective:**

This study aims to investigate the relationship between RNs and hospital-based medical specialties staffing levels with inpatient COVID-19 mortality rates.

**Methods:**

We relied on data from AHA Annual Survey Database, Area Health Resource File, and UnitedHealth Group Clinical Discovery Database. In phase 1 of the analysis, we estimated the risk-standardized event rates (RSERs) based on 95,915 patients in the UnitedHealth Group Database 1,398 hospitals. We then used beta regression to analyze the association between hospital- and county- level factors with risk-standardized inpatient COVID-19 mortality rates from March 1, 2020, through December 31, 2020.

**Results:**

Higher staffing levels of RNs and emergency medicine physicians were associated with lower COVID-19 mortality rates. Moreover, larger teaching hospitals located in urban settings had higher COVID-19 mortality rates. Finally, counties with greater social vulnerability, specifically in terms of housing type and transportation, and those with high infection rates had the worst patient mortality rates.

**Conclusion:**

Higher staffing levels are associated with lower inpatient mortality rates for COVID-19 patients. More research is needed to determine appropriate staffing levels and how staffing levels interact with other factors such as teams, leadership, and culture to impact patient care during pandemics.

## Introduction

COVID-19 is a serious infection that spreads rapidly within a population and has placed tremendous strain on hospitals worldwide. According to the CDC, as of May 2022, around 83 million people were infected with COVID-19, and a million lost their lives in the U.S. alone [1]. COVID-19 is classified as a B-CoVs, and rats and bats are identified as the main gene sources for B-CoVs [2]. While 80% of individuals infected with the virus exhibit mild symptoms, around 20% produce a “hyperinflammatory response” and suffer from severe respiratory distress [3, 4]. Complications and hospitalization rates vary between individuals. Complication rates are around 45% for individuals with diabetes, cancer, hypertension, or cardiovascular diseases [5].

Hospitals faced unprecedented circumstances and pressures during the COVID-19 pandemic, with documented variations in COVID-19 caseloads and occupancy rates [6]. Few studies, however, reported mortality rates at the hospital level. Roth et al. reported an average hospital mortality rate of 15.8% for March through November 2020 [7]. Most studies relied on patient characteristics to explain variations in COVID-19 mortality at the hospital level. For instance, previous studies found that patients who were older experienced higher COVID-19 mortality rates [8, 9]. In addition to age, higher mortality rates were reported among COVID-19 patients with chronic diseases and dyspnea, and those admitted to the hospital with out-of-range laboratory results upon admission [9]. While patient characteristics helped explain some of the variability in mortality rates, hospital characteristics and county-level factors are likely also to have influenced COVID-19 mortality rates.

Strained capacity limited hospitals in their response to any "pandemic-associated surge" and contributed to mortality from COVID-19 [10]. Data from the epicenter of the pandemic provided evidence that there was variation in mortality rates even within a single region [11]. This variation can be explained by the impact the availability of healthcare resources has on COVID-19 mortality rates [11]. Limited research examined hospital-level predictors of COVID-19 mortality. As Asch et al. argue; “hospital-level mortality may depend not just on patient risk factors, but also on the hospital where patients are admitted” [12]. Traditional disaster planning in the United States focused on the surge experienced by emergency departments during disasters [13]. However, during pandemics, such as the current COVID-19 pandemic, general/surgical units, and ICUs are also overwhelmed. When pandemics occur, hospitals face a significant increase in demand in a short period of time which has an impact on patient outcomes. Demand surge is defined as a “sizable increase in demand of medical or public health resources” [14]. During public health emergencies, such as pandemics, capacity, specifically beds, staff, and equipment experience “severe strain resulting from patient surges” [15]. There are four key elements, referred to as the 4S, to capacity: 1) staff, 2) stuff (beds and equipment), 3) structure (space), and 4) management systems [16, 17]. The availability of hospital beds and equipment in addition to sufficient staffing levels are essential elements of any response to increased demand in healthcare [18].

Staffing capacity impacts the workload experienced by clinical staff which is associated with patient outcomes [19]. Staffing levels in healthcare organizations are associated with quality of care and patient experience [19]. Staffing levels impact mental and physical workload and, consequently, patient outcomes [19]. Heavy mental workload, specifically, is associated with delayed information processing [20] and fatigue [21], both of which contribute to medical errors. The relationship between lower nurse staffing levels and higher mortality rates is well-documented in the literature [22, 23]. Moreover, lower staffing levels contribute to hospital acquired infections [24], and, therefore, might contribute to the spread of the SARS-CoV 2. To emphasize the importance of appropriate staffing levels and minimize the risks associated with low staffing levels,the state of California for example, mandated minimum RN staffing levels. In addition to the relationship between staffing levels and patient outcomes, it is important to account for the fact that pandemics impact sick absenteeism [25]. The burdens of pandemics also have a psychosocial effect on health care staff [26]. For example, nursing staff might have to take on new roles to meet the surge in demand. Recent research demonstrates that hospital staff experienced burnout during the peak of the pandemic [27, 28]. Low staffing levels are among the critical predictors of staff burnout [24]. In conclusion, staffing is a critical determinant of the ability of hospitals to respond effectively to the increased demand during a pandemic.

Studies in the United States on COVID-19 patient outcomes were limited by the following factors (1) datasets containing minimal clinical information, (2) analysis that included a limited number of hospital-level variables, or (3) findings based on data from only one healthcare facility [29]. More research is needed with analysis that includes a representative sample of hospitals in the U.S. and incorporates a more comprehensive list of hospital-level variables. Moreover, more research is needed to understand the relationship between staffing levels and patient outcomes during pandemics. Our study, therefore, contributes to the literature by: (1) including a broader set of hospital level variables, (2) including a national sample of hospitals in the U.S., and (3) incorporating staffing levels in our model as a predictor of COVID-19 mortality rates. In summary, the objective of this paper is to study the association between hospital staffing levels and county level variables with COVID-19 patient outcomes at the hospital level. We focus on staffing levels of registered nurses (RNs), hospitalists, emergency medicine physicians, and intensivists (staff). This paper builds on and expands the model used by Asch et al. [12] who only examined a limited set of hospital level variables over a shorter period of time.

## Methods

### Data sources

We used de-identified administrative claims from the UnitedHealth Group Clinical Discovery Database for both Medicare and commercial fully insured populations (including patients with administrative services only (ASO) accompanied by a daily record of hospital admissions due to primary or secondary diagnosis of COVID-19 (primarily Alpha, Beta, Gamma, or other subvariants prevalent during the study period) along with the daily disposition status (admitted, discharged, transferred, or expired) available until December 31, 2020. To create the study population, we included all Medicare Advantage and commercially insured hospitalized patients, including data for ASO plan members where available in the database. Next, we restricted the sample to patients continuously enrolled for at least six months to capture better the claims-based historical comorbidities. We further confined the sample to patients who are older than eighteen and excluded any cases with missing patient-level risk factors. To accommodate two pandemic surges that happened in 2020, we excluded patients admitted before March 1, 2020 and after December 31, 2020. We included hospitals (not‐for‐profit, for‐profit, and nonfederal public general hospitals) only with medical provider numbers matched to the 2020 American Hospital Association (AHA) Annual Survey Database. Patients with readmission or a history of hospital transfer within 30-days of the initial admission were precluded to evade any potential misattribution of hospital-level outcomes; a sensitivity analysis relaxing this criterion was also performed. To improve the robustness of hospital-specific parameter estimates, we included sites with at least ten patients who were present in our database; see S5 Fig in S1 File for details. This study was reviewed and deemed exempt by the institutional review board of UnitedHealth Group.

We used hospital‐level data from the 2020 AHA Annual Survey Database and county-level data from the 2019–2020 Area Health Resource File (AHRF). We obtained 2018 Social Vulnerability Index (SVI) from the Centers for Disease Control and Prevention/ Agency for Toxic Substances and Disease Registry (CDC/ATSDR). We also obtained county-level cumulative COVID-19 case rates for March 1 to December 31, 2020 from *The New York Times* database.

### Dependent variable

To represent an outcome close to a 30-day hospital mortality rate, hospital’s risk standardized event rate (RSER) was used. This estimated measure reflects a composite of either inpatient mortality or referral to hospice within 30 days of hospitalization due to COVID-19—based on the National Quality Forum-approved hierarchical generalized linear models accounting for heterogeneity [30], RSERs were adjusted for patient-level characteristics (e.g., demographics, Elixhauser comorbidities [31], community or nursing facility admission), hospital size measured by the total facility admissions reported in 2020 AHA Annual Survey Database and discretized time points featuring different pandemic phases with respect to the differences between patients’ first hospital admission and March 1, 2020.

### Predictors

#### Hospital-level variables

The primary objective of this paper was to study the association between hospital staffing levels and county-level variables with COVID-19 mortality rates. We captured the staffing levels of RNs (full-time equivalent (FTE) RNs divided by the number of hospital beds), hospitalists (FTE hospitalists divided by the number of medical/surgical beds), medical/surgical intensivists (FTE medical/surgical intensivists divided by the number of medical/surgical beds), and emergency medicine physicians (total privileged emergency medicine physicians divided by the number of emergency department visits) in 2020.

#### County-level variables

At the county level, we use the Social Vulnerability Index (SVI) to examine the relationship between county-level factors and COVID-19 mortality rate. The pandemic disproportionately impacted socially vulnerable populations since social and environmental factors influence a community’s ability to withstand disasters like pandemics [32]. The CDC’s SVI groups 15 indicators into four themes and ranks counties on the four themes and an overall SVI score. The four themes are: socioeconomic status (Theme 1), household composition and disability (Theme 2), minority status and language (Theme 3), and housing type and transportation (Theme 4). The themes are represented by percentiles, with higher values indicating greater vulnerability. Previous research indicates that there is a significant relationship between SVI and both infection and vaccination rates [32, 33]. Therefore, we used them in the regression model to examine the relationship between a county’s standing on the four themes and COVID-19 mortality at the hospital level.

### Covariates

We limited our study to general acute hospitals. According to Donabedian’s structure-process-outcomes framework, structural characteristics such as type of hospital ownership, staffing, and size impact the delivery of health services [34]. Therefore, at the hospital level, we control for size, ownership (not‐for‐profit, for‐profit, and local public hospitals), whether a hospital has one or more Accreditation Council for Graduate Medical Education (ACGME) accredited programs, Medicare and Medicaid share of total inpatient days, baseline occupancy rate (calculated as the number of inpatient days divided by 365 multiplied by the number of hospital beds in 2020), and whether a hospital is Magnet recognized. For hospital size and bed capacity, we included the number of hospital beds, medical/surgical intensive-care unit (ICU) beds, and airborne infection isolation rooms. We controlled for Magnet status since it is an indicator of organizational leadership and culture that fosters high‐quality care. At the county level, we controlled for location (urban vs. rural county) market concentration (measured by Herfindahl‐Hirschman Index (HHI), which is calculated by summing the square of the market share of total admissions in a county for each hospital in the county), and the cumulative COVID case rate per 10,000 residents during March-December 2020.

### Statistical analysis

Hierarchical model is fitted to estimate the odds of mortality or referral to hospice adjusting for the selected covariates including Elixhauser based comorbidities, demographic variables (age and gender), status of transfer from nursing facility admission, the number of days between each hospital admission and March 1, 2020, and the volume of average patient admissions in 2020; the last variable is to adjust for the association between hospital volume and mortality rates. Variation among patients is accounted for through hospital specific random effects which are estimated via restricted maximum likelihood (REML). We report the adjusted odds ratios of each risk factor with 95% confidence intervals along with p-values based on Wald test statistics. Each hospital’s RSERs were calculated via recycled prediction where the idea is to take average over all patients’ predicted probabilities of experiencing events had each of them hypothetically been treated at each hospital; for details we refer to Asch, et al., George, et al. [35], Silber, et al. [36–38]. Second model is fitted by excluding patients with readmission or transfer to facilities that are only short-term, long-term, and critical access care; Kendall rank correlation coefficient was used to compare the differences in ranking of hospitals with respect to the corresponding RSERs. Technical details are provided in the Supporting information.

We utilize the estimated RSER as the dependent variable in the subsequent model to investigate the association between hospital staffing levels and county-level variables with COVID-19 mortality rates. Since RSER is a proportion, we use beta regression [39, 40]. We also include state-level fixed effects in the model to account for any unobserved heterogeneity among the states. We use bootstrapping (n = 1,000) to estimate the 90% and 95% confidence intervals of the parameter estimates. All analyses were conducted using R version 3.6.3.

## Results

We analyzed data in two phases. In Phase 1, the patient-level analysis was based on 1,398 hospitals exploiting 95,915 patients. Descriptive statistics of the patient-level variables are stipulated in S2 Table in S1 File. Adjusted ORs associated with the risk factors that were used in calculating RSERs are illustrated in S1 File. Information on the estimated RSERs can be found in S1 File in an ascending order with the corresponding interquartile range (IQR); here smaller RSER indicates lower rate of experiencing events at hospital-level.

In Phase 2, we first removed hospitals that had missing values in county-level variables and left with 1,364 hospitals. Seven states (Delaware, District of Columbia, New Mexico, North Dakota, South Dakota, Vermont, West Virginia, and Wyoming) have each fewer than five hospitals with reported data, with a total of 19 hospitals. Since we had state-level fixed-effects in the model, we excluded hospitals from the seven states due to their small number of observations and obtained the final analysis set of 1,345 hospitals. In the final sample, only one hospital-level predictor, hospitalist staffing level (as will be detailed later), had missing values. The 243 missing values were replaced by the median value of that variable. Descriptive statistics of the hospital-level variables are summarized in Table 1.

**Table 1:** Summary statistics of hospital and county level variables.

Variable	Mean (Standard Deviation) or Percentage of Samples
Dependent Variable	
Risk standardized event rate (RSER)	10.78 (2.96)
Hospital-Level Variables	
RN FTEs per hospital bed	2.06 (0.78)
Hospitalists per medical/surgical bed	0.16 (0.34)
Intensivists per medical/surgical ICU bed	0.28 (0.63)
Respiratory therapists per hospital bed	0.096 (0.046)
Emergency physicians per ER visit	0.00074 (0.00093)
Number of hospital beds	298.91 (254.90)
Number of medical/surgical ICU beds	21.09 (23.65)
Number of airborne infection isolation rooms	23.49 (32.22)
Ownership:	
• Not-for-profit	70.75%
• For-profit	17.96%
• Local public	11.29%
Has one or more ACGME accredited programs (binary)	63.71%
Magnet recoganized (binary)	19.21%
Baseline occupancy rate (%)	59.64 (15.41)
Medicare share of inpatient days (%)	51.58 (11.20)
Medicaid share of inpatient days (%)	20.12 (9.25)
County-Level Variables	
Urban county (binary)	85.85%
HHI	0.090 (0.157)
COVID cumulative case rate per 10,000 residents during March-December 2020	636.30 (196.82)
Sample size	1,345

The parameter estimates, p values, and the 90% and 95% confidence intervals based on non-parametric bootstrapping (n = 1,000) for the hospital-level beta regression model are shown in Table 2. Based on the diagnostic plots of the regression model, the model assumptions were largely met. All variance inflation factors (VIFs) were less than 4.04, which suggested that multicollinearity was not a concern. The Wald test rejected the null hypothesis that all coefficients were simultaneously equal to zero. The pseudo R-squared of the model is 0.236.

**Table 2:** Parameter estimates quantifying the association between RSERs and hospital- and county-level variables.

			
*Variables*	*Estimates*	*P Value*	*90% CI*	*95% CI*
(Intercept)	-2.181	<0.001	(-2.282, -2.071)	(-2.305, -2.052)
RN	-0.017	0.067	(-0.032, -0.002)	(-0.036, 0.001)
Hospitalists	-0.006	0.541	(-0.021, 0.005)	(-0.026, 0.008)
Intensivists	-0.005	0.529	(-0.018, 0.007)	(-0.020, 0.010)
Emergency physicians	-0.019	0.035	(-0.033, -0.004)	(-0.037, -0.002)
# hospital beds	0.030	0.037	(0.003, 0.056)	(0.000, 0.062)
# medical/surgical ICU beds	0.000	0.999	(-0.021, 0.022)	(-0.025, 0.025)
# airborne infection isolation rooms	-0.004	0.684	(-0.022, 0.011)	(-0.024, 0.014)
Not‐for‐profit (reference)				
For-profit	-0.040	0.075	(-0.080, -0.003)	(-0.088, 0.003)
Local public	-0.010	0.690	(-0.056, 0.037)	(-0.066, 0.048)
One or more ACGME programs	0.062	0.001	(0.031, 0.094)	(0.025, 0.101)
Magnet	-0.031	0.144	(-0.067, 0.002)	(-0.072, 0.008)
Occupancy rate	0.030	0.002	(0.013, 0.046)	(0.010, 0.049)
Medicare share	0.013	0.220	(-0.003, 0.031)	(-0.006, 0.033)
Medicaid share	0.015	0.184	(-0.004, 0.034)	(-0.008, 0.037)
Urban county	0.096	<0.001	(0.050, 0.147)	(0.042, 0.155)
HHI	0.005	0.548	(-0.010, 0.020)	(-0.014, 0.023)
SVI Theme 1	-0.001	0.918	(-0.024, 0.019)	(-0.027, 0.023)
SVI Theme 2	0.010	0.363	(-0.008, 0.028)	(-0.011, 0.032)
SVI Theme 3	-0.012	0.205	(-0.029, 0.003)	(-0.033, 0.007)
SVI Theme 4	0.016	0.099	(0.000, 0.033)	(-0.003, 0.036)
COVID case rate	0.033	0.003	(0.014, 0.053)	(0.009, 0.058)

Reported are the point estimates, p-values (at 5% level of significance), and 90% and 95% CIs generated by bootstrapping. All continuous independent variables are centered and scaled.

Among the four staffing variables, higher levels of RNs (estimate: -0.017; 90% CI: [-0.032, -0.002], 95% CI: [-0.036, 0.001]) and emergency medicine physicians (estimate: -0.019; 90% CI: [-0.033, -0.004], 95% CI: [-0.037, -0.002]) staffing levels were associated with lower RSERs at the 10% and 5% (marginally for RN) significance level respectively, suggesting that these two types of medical personnel helped to reduce COVID-19 mortality rate on average. Possible values for the true mortality rate decrease with respect to RN that were most compatible with our data, given our statistical model, ranged from -0.032 to -0.002 (90% CI).

At the county level, urban counties had higher mean COVID-19 mortality rates than rural counties (estimate: 0.096; 90% CI: [0.050, 0.147], 95% CI: [0.042, 0.155]) signaling statistical significance at both 5% and 10% significance level. Higher SVI Theme 4 (housing type and transportation) value was associated with higher mortality rate, on average, at the 10% (but not 5%) significance level (estimate: 0.016; 90% CI: [0.000, 0.033], 95% CI: [-0.003, 0.036]). Specifically, housing types of multi-unit structure, mobile home, and group quarter, which usually resulted in higher population density, and no personal vehicle for transportation contributed to higher SVI Theme 4 values (i.e., higher level of vulnerability).

Regarding the covariates, for-profit hospitals had lower mortality rate, on average, compared to not-for-profit hospitals (estimate: -0.04; 90% CI: [-0.080, -0.003], 95% CI: [-0.088, 0.003]) signaling statistical significance at 10% but not at 5%; possible values for the true mortality rate decrease with respect to profit-status that were most compatible with our data, given our statistical model, ranged from -0.080 to -0.003 (90% CI).

Hospitals having one or more ACGME accredited programs (estimate: 0.062; 90% CI: [0.031, 0.094], 95% CI: [0.025, 0.101]) tended to have higher mortality rates on average compared to hospitals not having such programs. As for hospital size, higher number of hospital beds (estimate: 0.030; 90% CI: [0.003, 0.056], 95% CI: [0.000, 0.062]) was associated with higher mortality rate; possible values for the true mortality rate increase that were most compatible with our data, given our statistical model, ranged from 0.003 to 0.056 (90% CI). Hospital baseline occupancy rate was positively and significantly correlated with COVID-19 mortality rate, indicating that hospitals with higher occupancy rates tended to have worse patient outcomes in terms of survival (estimate: 0.030; 90% CI: [0.013, 0.046], 95% CI: [0.010, 0.049]). Finally, hospitals located in counties with higher cumulative COVID-19 case rate had higher mortality rate (estimate: 0.033; 90% CI: [0.014, 0.053], 95% CI: [0.009, 0.058]) highlighting statistical significance at both 5% and 10% level of significance; possible values for the true mortality rate increase that were most compatible with our data, given our statistical model, ranged from 0.014 to 0.053 (90% CI).

## Discussion

Based on the regression results, COVID-19 patients receiving treatment in larger not-for-profit teaching hospitals with lower RN and ER physicians staffing levels had the worst outcomes. Moreover, teaching hospitals and hospitals with higher occupancy had higher COVID-19 mortality rates. In terms of county characteristics, hospitals located in urban areas and counties with higher COVID-19 infection rates had a lower likelihood of surviving. Moreover, counties with greater social vulnerability, specifically in the Housing Type and Transportation theme, had higher mortality rates. Note that the strengths of association with mortality rate among RN, for-profit hospital status, and SVI theme 4, while were biologically implicative, were not as potent as other confounders. Bootstrapping provides better uncertainty quantification for point estimates, however this does not necessarily alleviate the effects of unmeasured confounders on an association model which may play a role in inferences.

Staffing levels matter! The positive relationship between RN staffing levels and patient outcomes is well-documented in the literature [19, 41, 42]. However, there is limited research on how staffing levels impact patient outcomes during pandemics. According to our knowledge, recent research examined the relationship between nurse staffing levels and COVID-19 infection and mortality rates in nursing home settings but not in hospital settings. Harrington et al. found a negative association between nurse staffing levels and infection rates in nursing homes [43]. In another study, total nursing hours were associated with fewer deaths and a lower likelihood that the nursing home would experience an outbreak [44]. Our study extends the literature on RN staffing and COVID-19 outcomes to include hospital settings. We found a marginally significant positive relationship between RN staffing levels and lower COVID-19 mortality rates. Hospital administrators need to evaluate the heavy workload RNs experience during pandemics. Staffing levels and consequently workload not only predict patient outcomes but has a direct impact on healthcare workers well-being [45]. Ensuring adequate RN staffing levels protects the well-being of our nursing workforce by preventing heavy workloads and easing the psychological stress RNs experience when simultaneously dealing with a surge in demand and the fear of carrying an infectious disease back home to their families and friends [46].

According to our analysis, ER physician staffing is also associated with better COVID-19 patient outcomes. Patients with severe COVID-19 infections are admitted to hospitals through the ER, where they are first treated and triaged by ER physicians. Therefore, ER physicians, particularly, are critical in the fight against morbidity and mortality from the novel coronavirus since they are the first point of contact. As Gaeta and Brennessel argue “physicians serve as a unique resource connecting a diverse patient population with emergent management of conditions” [47]. Emergency department staff are responsible for triaging patients and for managing those patients with and without confirmed infection, all while being at the risk of contracting and spreading the virus and feeling burned out because of the long work shifts [48]. Our study found a significant positive relationship between emergency medicine staffing levels and lower COVID-19 mortality rates. This finding is important since there is limited research on the association between emergency medicine physicians and patient outcomes under normal circumstances and during pandemics. We did not find any significant relationship between hospitalists and intensivists staffing levels and COVID-19 patient outcomes. This could be partly due to the lack of data on the most appropriate output measures to use for hospitalist and intensivist staffing levels. While the number of ER visits was available, the number of ICU admissions or inpatient days, which would be a more accurate output for intensivists, and the number of medical/surgical admissions or inpatient days, which would be a more accurate output for hospitalists, were not available.

Donabedian argued that hospital structural attributes impact patient outcomes [49]. In addition to staffing levels, our analysis indicates that hospital ownership, size, and teaching status are associated with COVID-19 patient mortality rates. For-profit ownership was associated with better COVID-19 patient outcomes. The health services literature does not provide concluding evidence to support differences in patient outcomes between for-profit and not-for-profit hospitals. Some scholars have argued that ownership does not result in a meaningful difference between hospitals [50]. Quality is not influenced by ownership but rather by institutional context, market conditions, services provided by the hospital and other factors [51, 52] To provide more insights into hospital ownership and performance, a study focused on comparing the performance of hospitals before and after they converted from NFP to FP ownership [53]. Converted hospitals reported better financial performance after changing their ownership to FP; however, no significant change occurred in quality and patient outcomes [53]. While our finding that for-profit hospitals had better COVID-19 outcomes is surprising, other studies have a found better patient outcomes at for-profit hospitals [54]. For-profit hospitals have more financial resources, enhancing their ability to hire personnel and purchase equipment and supplies [53, 54]. This, in addition to differences in teaching status, geography, and services offered, could help explain the differences in COVID-19 patient mortality rates between not-for-profit and for-profit hospitals.

As for teaching hospitals, previous research offers conflicting findings on the relationship between teaching status and patient outcomes [55, 56]. Hoyer et al. argue that academic medical centers serve populations with more complex healthcare needs and have a larger share of patients with low socioeconomic status [56]. Therefore, academic medical centers fare worse on certain quality indicators, such as hospital-wide readmission rates [56]. The same reasoning can be applied to explain the worst mortality rates at teaching hospitals. Our findings also indicate a positive association between hospital size and occupancy rate with worst COVID-19 outcomes. Our findings are consistent with previous research that found worse RSERs at medium to large hospitals [12]. Larger hospitals might find it more challenging to standardize processes and care protocols which can impact patient outcomes. Moreover, high occupancy rates indicate strained resources, which is likely to impact patient outcomes.

Greater social vulnerability, COVID-19 infection rates, and urban settings were the county-level variables associated with worst RSERs. According to our findings, housing structures, crowded housing, and lack of transportation means, which result in greater social vulnerability, are associated with worst COVID-19 outcomes. Our finding is consistent with previous research that found "poor housing conditions and transport accessibility" are associated with higher COVID-19 case fatality [57]. Previous research also supports our finding that urban counties which tend to have higher population density and larger household size have higher mortality rates. Chandra et al., for instance, found a positive relationship between population density and mortality from the influenza pandemic of 1918–1919 [58]. Tamblyn argue that communities with high population densities are more likely to experience outbreaks [59]. Hospitals in counties with higher COVID-19 infections experience more severe demand surges which strains the local healthcare system and thus experience higher mortality rates.

This study is not without limitations. Our sample is limited to patients whose outcomes are reported in the UnitedHealth Group Clinical Discovery database which, for example, specifically does not include those patients who are insured via Medicaid. Moreover, since we do not have reliable race data for the commercially insured patient population, we used residential level information to determine the distribution of patients by race at the hospital level. In addition, while we include 2020 staffing levels, our data is annual and does not include specific staffing levels during surges in demand. While our study has strong observarions, we acknowledge that our study is rarely sufficient to establish robustness of our findings for which a thorough investigation is warrented.

## Conclusion

Our study is the first to examine the association between RN and physician staffing levels with hospital-level COVID-19 mortality and includes a large geographically diverse cohort over a significant time span. Based on our findings, staffing levels are key predictors of patient outcomes. While policymakers have focused on increasing bed capacity, the supply of personal protective equipment, and ventilators, emergency preparedness plans and future policies should also take into account RN and ER physicians staffing levels. More research is needed to determine appropriate staffing levels during pandemics and how staffing levels interact with other factors such as teams, leadership, and culture to impact patient care. This will help hospitals establish benchmarks, policies and plans to handle demand surge during pandemics, given the impact staffing levels have on patient outcomes.

## Supporting information

S1 File Additional information about the methodologies, technical details, results, analytical dataset, and data definitions. (DOCX)
