# Supplementary material for: Is there an association between hospital staffing levels and inpatient-COVID-19 mortality rates?
Source: PLoS One. 2022 Oct 19;17(10):e0275500. doi: 10.1371/journal.pone.0275500 (PMC9581383; doi:10.1371/journal.pone.0275500)
Supplement: S1 File — (DOCX) [file pone.0275500.s001.docx]

**S1 File.** Additional information about the methodologies, technical details, results, analytical dataset, and data definitions.

## S1A. Hospital Specific Risk Standardized Event Approach

Let $i$ denote hospital and $j$ hospitalized patient associated with COVID diagnosis such that $i = 1, \ldots, I$ and $j=1,\ldots,n_{i}$, where $I$ is the total number of hospitals and $n_{i}$ is the total number of patients with COVID-related hospitalization. Let N = $\sum_{i=1}^{I} n_{i} be the total number of patients.$Let$Y_{ij}$ refer to the status of the $j$-th patient taking a value either 1 if a patient is deceased or transferred to hospice within 30-day of hospital admission date, or 0 otherwise. Denote by $\mathcal{X}_{ijk}$ the $k$-th covariate (e.g., gender, age, status of transfer from a nursing facility and Elixhauser comorbidities, discretized bins of time-differences); here $k = 1, \ldots, K$ with$K$ denoting the total number covariates.

In our application, we perform the analyses without variable selection. However, though not reported, we use a univariate association filter approach to select influential features. The idea is to fit a series of univariate generalized linear models (GLM) with a logit link function corresponding to each variable of interest. We performe hypothesis tests for null effects via Wald test statistics, compared p-values, and selected the ones that are less than a prespecified threshold of 0.10. Three Elixhauser based comorbidities - liver disease, peptic ulcer, and rheumatoid arthritis are excluded as the corresponding p-values were greater than 0.10. While more formal procedures like regularization penalty based methods can be adopted, this necessitate to adjust for post-selection inference. We report the results in the paper without variable selection as results were similar. Numerical variables such as age and time differences between hospital admissions and March 1, 2020, are categorized into bins for ease of interpretability throughout the study. With an abuse of notation, denote by $X_{ijk}$ the $k$-th candidate covariate for the multivariate analysis that is to be discussed next; i.e$. X\subseteq\mathcal{X.}$

Hospital specific risk standardized event rates (RSER) are estimated using a hierarchical model (which is also known as generalized linear mixed model (GLMM)) using the procedures described in Asch, et al.9 A fixed effect for logarithm of hospitals’ volume (i.e., average number of patients admitted in 2019-20) is added to account for potential variability in patient case-mix distribution among different sized hospitals. Alternatively, the volume of hospitals can represent the total number of COVID admitted patients (who are continuously enrolled for at-least 6 months) in the sample. The key difference between these two measures is that the former captures the overall size of hospital while the latter signals only the COVID admitted (and insured) patients. We observe moderate correlation (Pearson correlation coefficient 0.46 with 95% confidence interval (0.41, 0.50), p-value < 0.0001) between these two measures; and a sensitivity analysis is performed to estimate RSER using this measure.

A multivariable GLMM framework, as below, is fitted

$${logit Pr(Y}_{ij}=1)=\beta_{0}+\sum_{k=1}^{K} X_{ijk}\beta_{k} + \gamma log({vol}_{i})+ b_{i0}\text{.}$$

Here fixed effect parameters, denoted by $\boldsymbol{\beta}= {(\beta}_{0}, \beta_{1},\ldots,\beta_{K},\gamma)$, quantify the effects of covariates on likelihood of experiencing events and characterize the deviation from the overall mean effect. Random effects $b_{i0}$ are assumed to follow Gaussian distribution with mean $0$and unknown variance $\sigma_{0b}^{2}$. From a hierarchical point-of-view, this model can be expressed as below

$${logit Pr(Y}_{ij}=1)= \mu_{i}+\sum_{k=1}^{K} X_{ijk}\beta_{k}\text{,}$$

$$\mu_{i}= \beta_{0}+\gamma log\left( {vol}_{i} \right)+ b_{i0}.$$

where $\mu_{i}$ represents hospital-specific mean.

Using the similar intuition to recycled predictions, RSERs are computed by averaging the event rates for all $N$ = $\sum_{i=1}^{I} n_{i}$ had each one of them been treated hypothetically in every hospital. We stack predicted probabilities in an $N\times I$ dimensional matrix and take column-wise average to obtain $I$ RSERs. Denote by $\tau$ a different hospital from $i$. Define the probability of experiencing the event if the $j$-th patient is coming from the $\tau$-th hospital

${p_{\tau j}=E(Y}_{\tau j}=1\left| X_{ij},{{vol}_{\tau}, b}_{\tau0} \right) = 1/\{1 + exp$(-$\beta_{0}-\sum_{k=1}^{K} X_{ijk}\beta_{k} - {\gamma log\left( {vol}_{\tau} \right)-b}_{\tau0})\}$.

Next, RSER for the $i$-th hospital is computed as

$$s_{i,DS}\text{ = }\sum_{\tau=1}^{I} \sum_{j=1}^{n_{\tau}} p_{\tau j} / N.$$

Such recycled event rates are also known as standardized event rates and entail probabilistic attribute.

Fixed and random effects are estimated using restricted maximum likelihood (REML) approach where the likelihood function is evaluated via adaptive Gauss-Hermite quadrature with 11 quadrature points and implemented by $GLMMadaptive$ R-package with default control parameters. The random effects are obtained as best linear unbiased predictors (BLUP) using a Bayesian formulation. We assessed the collinearity between variables via generalized variance inflation factor (GVIF) where the estimated GVIF are less than 2.00. We also fitted a multivariable GLM without random effects to check the appropriateness of using such random terms; the BIC are 61607.62 and 60801.17, and AIC are 61162.47 and 60549.52, respectively. REML estimate for the variance parameter associated with $b_{i0}$ is 0.22 with 95% CI (0.19, 0.26). Pearson’s correlation coefficient between the fixed effect estimated by GLM and GLMM is 1.00 (p-value < 0.0001). The corresponding measures for the goodness-of-fit are conditional $R$-squared value (0.25), marginal R-squared (0.20), $C$-statistic (0.72), and Somer’s $D_{xy}$ (0.44).

## S1B. Hospital Specific Risk Standardized Event Estimation

Figure SM1 displays the counts of analytical and sensitivity datasets. Figure SM2 illustrates the adjusted odds ratios (aORs) with 95% confidence intervals (CIs) of prognostic risk factors that were treated as fixed effects in the hierarchical model. Figure SM3 exhibits the risk standardized event rates (RSERs) in ascending order for 1,398 hospitals estimated via recycled predictions – the higher the value, the worse the hospital performance is. Figure SM4 represents the adjusted odds ratios with 95% confidence intervals for risk factors based on the hierarchical model where the total number of COVID admitted (and insured) patients per hospital (in the main sample) is used to represent the size of the corresponding hospital (sensitivity 1); Figure SM5 highlights the rank correlation between RSERs (%) computed from the models based on the original and the sensitivity dataset for the hospitals that are common in both datasets. Figure SM6 represents the adjusted odds ratio for a subset of dataset comprised of patients who 12 months of continuous enrollment in 2019 (sensitivity 2); Figure SM7 highlights the rank correlation among RSERs between main dataset and the sensitivity dataset. Figure SM8 depicts the association between the ranks of same hospital (among 1,398 hospitals) computed from the main analytical dataset and sensitivity dataset; the latter excludes patients who were transferred to short-term, long-term, and critical care access facilities. Figure SM9 compares prevalence of different risk factors across two dominant pandemic waves (i.e., March 1 – May 31, 2020 vs October 1 – December 31, 2020); here the analytical dataset is a subset of the original dataset including sites having at least five patients in each wave resulting in a cohort of 43,893 patients distributed across 861 hospitals. Figure SM10 shows the absolute and relative differences along with the percentage of improvements with respect to RSERs between early and late phase of the pandemic for each hospitals (Bland-Altman plot).

Table S1 details the data definitions and provides the corresponding ICD-10 codes. Table S2 provides summary statistics of patient-level data for the main analytical dataset.

S1 Fig. Waterfall counts for the study datasets used for phase-1 and phase-2 analyses


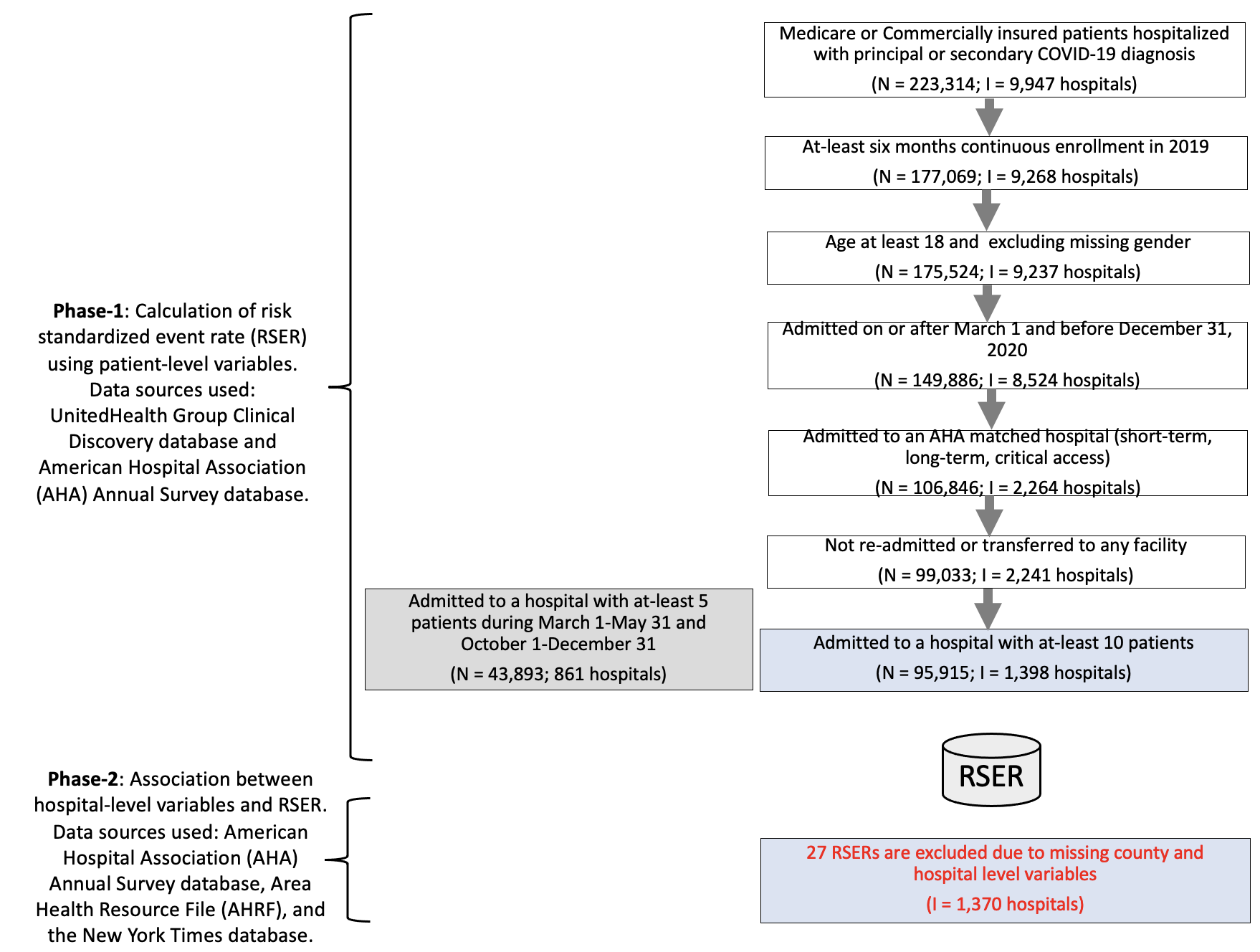


**Sensitivity-2**


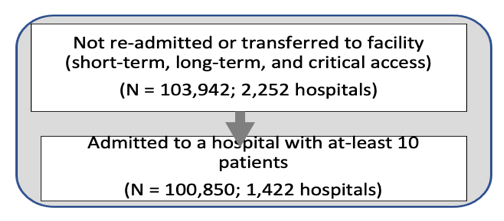


**Sensitivity-1**

Number of COVID hospitalization is used to represent the corresponding size of a hospital

**Sensitivity-3**


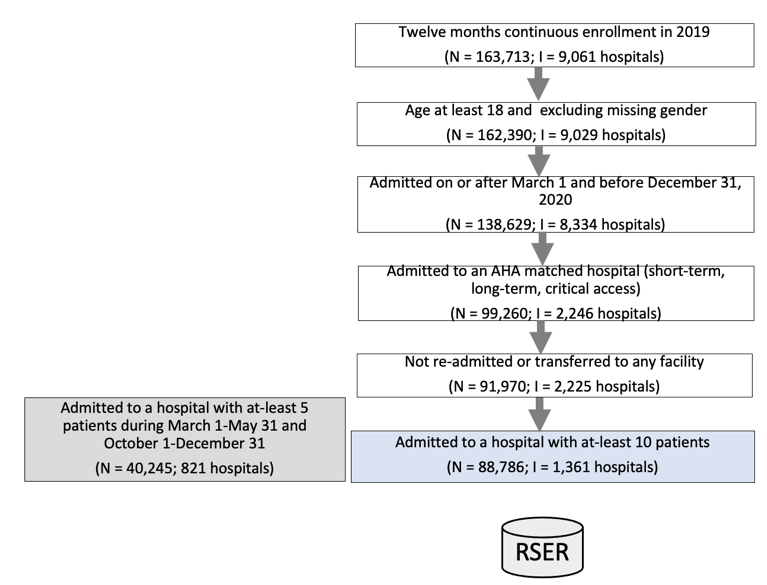


Please note that the shaded boxes with blue color were used in phase-1 and phase-2 analyses. Three sensitivity analyses were highlighted.

S2 Fig.Hierarchical model based adjusted odds ratios with 95% confidence intervals for risk factors; results are based on 95,915 patients admitted across 1,398 hospitals having at-least ten patients per site.


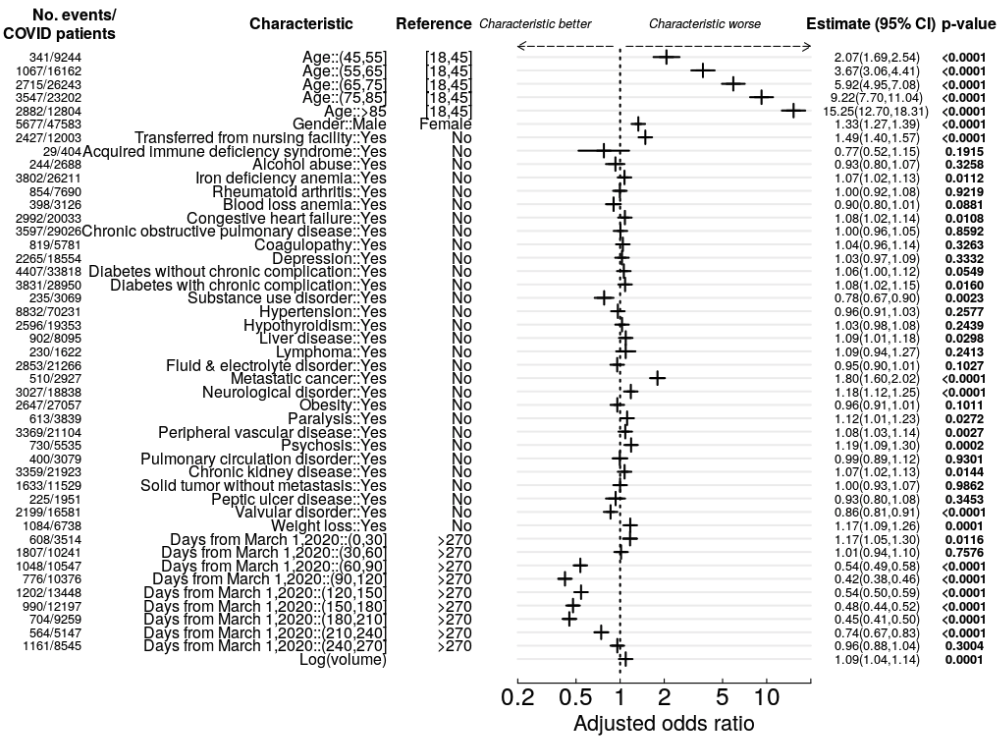


S3 Fig. Estimated RSERs for 1,398 hospitals based on the main analytical dataset with 95,915 patients; shaded area corresponds to inter quartile range (IQR) and dashed horizontal line refers to the observed event rate. Blue-colored “+” symbols represent hospitals with RSERs less than or equal to the observed event rate; and golden-colored “+” symbols highlight sites with RSERs greater than that.


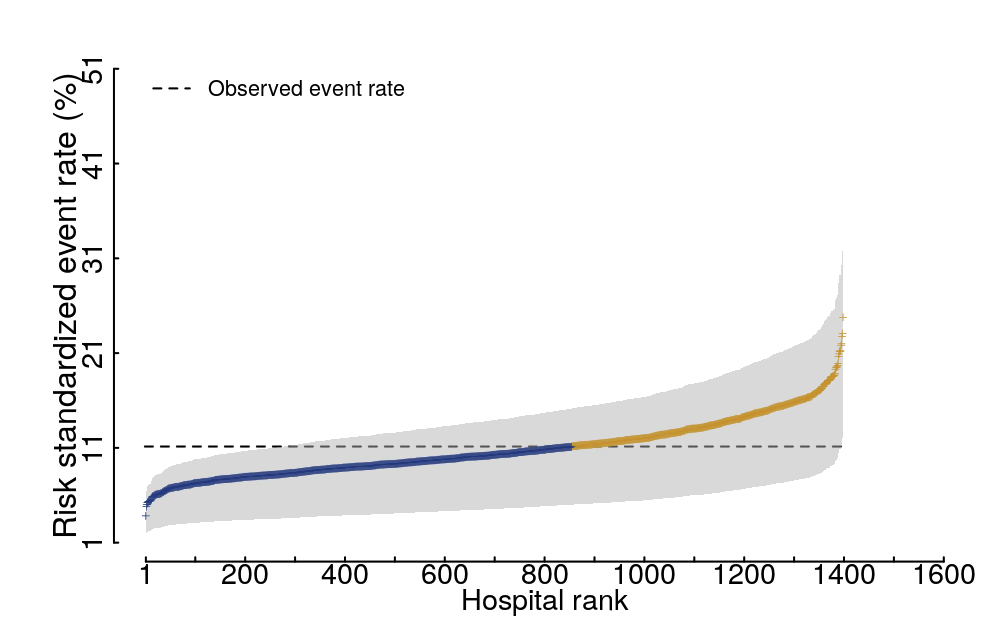


|  | RSER |
| --- | --- |
| Mean (95% CI) | 10.84 (10.68,10.99) |
| Q_0.50_  (IQR) | 10.27 (3.82) |
| Range | 20.96 |

S4 Fig. Adjusted odds ratios with 95% confidence intervals for risk factors with respect to the hierarchical model where total number of COVID admitted patients per hospital (in the main sample) is used to represent the size of the corresponding hospitals. Results are based on 95,915 patients admitted across 1,398 hospitals having at-least ten patients per site.


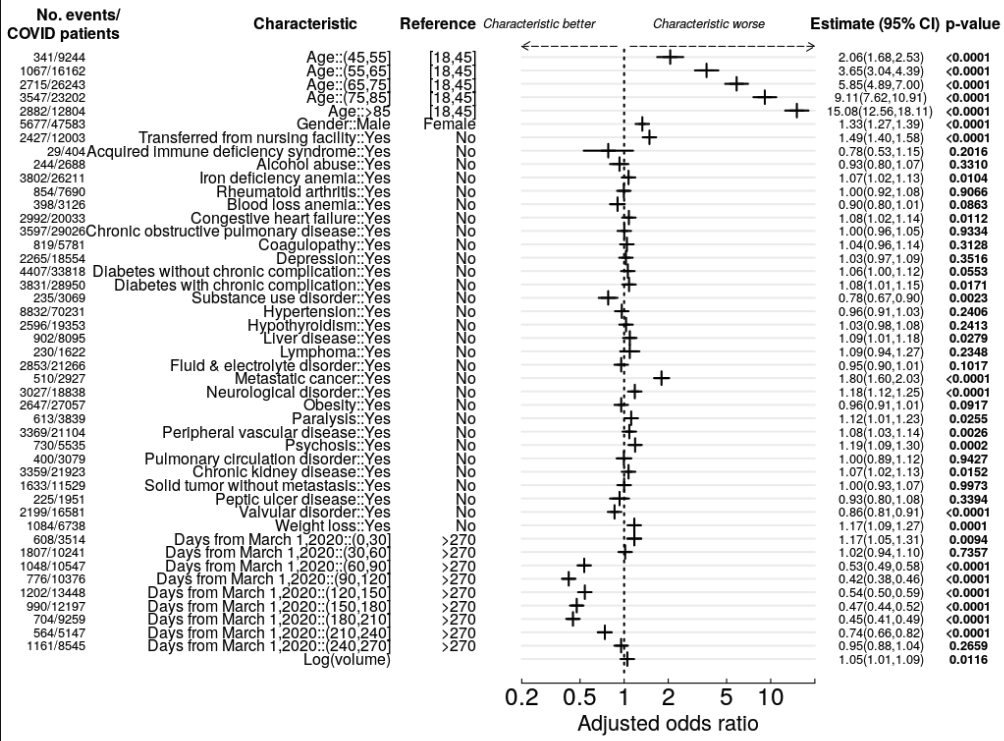


S5 Fig. (A) Association between RSERs (%) computed from the models based on the original and sensitivity dataset. Kendall rank correlation coefficient is reported for 1,398 sites’ RSERs based on two models where the first model (X-axis) refers to the main model that uses patients admitted in 2019-20 (as reported in 2020 AHA Annual Survey Database) to represent the size of a hospital where the second model (Y-axis) utilizes the number of COVID admissions to represent the size of a hospital. (B) Pearson correlation between these two measures – a smooth curve to data points is fitted to depict the underlying relatuonship.

(A)


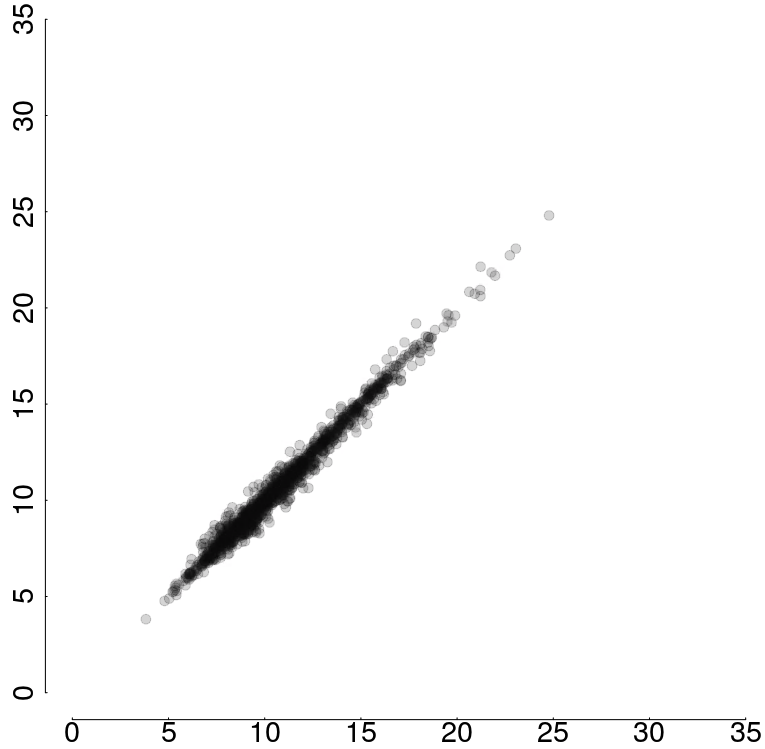


|  | N = 95,915;  I = 1,398 hospitals | N = 95,915;  I = 1,398 hospitals |
| --- | --- | --- |
| Mean  (95% CI) | 10.84  (10.68, 10.99) | 10.78  (10.61, 10.93) |
| Q_0.50_  (Q_0.25_,Q_0.75_) | 10.27  (8.69,12.51) | 10.19  (8.62,12.41) |
| Rank correlation | Kendall coefficient 0.90 (<0.00001) | |

RSER for sensitivity model-2

RSER for sensitivity model-1


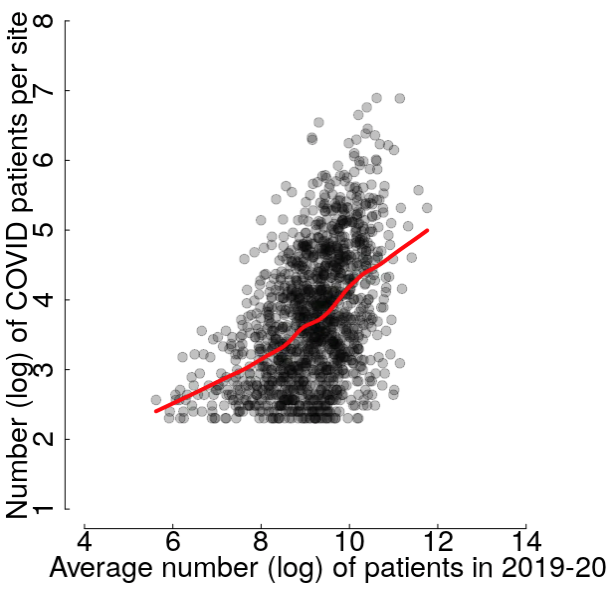


(B)

S6 Fig. Adjusted odds ratios with 95% confidence intervals for risk factors with respect to the hierarchical model. Results are based on 88,786 patients who were enrolled for the entire year of 2019 and admitted across 1,361 hospitals having at-least ten patients per site.


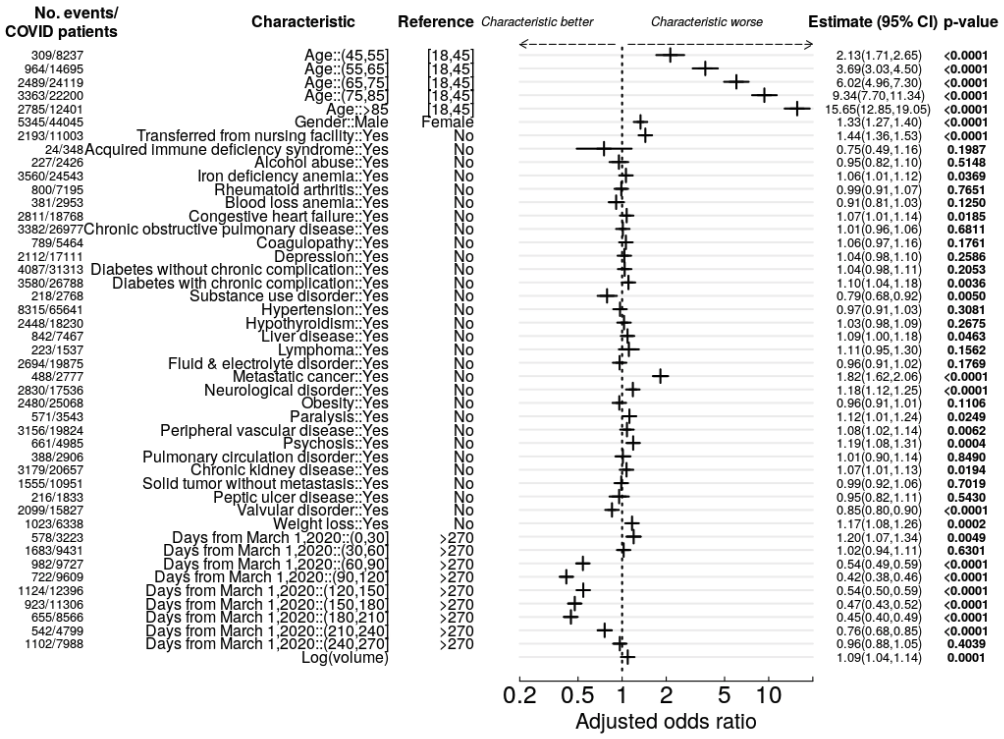


S7 Fig. Association between RSERs (%) computed from the models based on the original and sensitivity dataset. Rank correlation coefficient is reported for RSERs based on two models where the first model (X-axis) is based on the main analytical dataset where the second model (Y-axis) utilizes the subset of the main dataset comprised of patients with 12 months of enrollment in 2019. Displayed is the rank of common 1,361 hospitals between these two datasets.


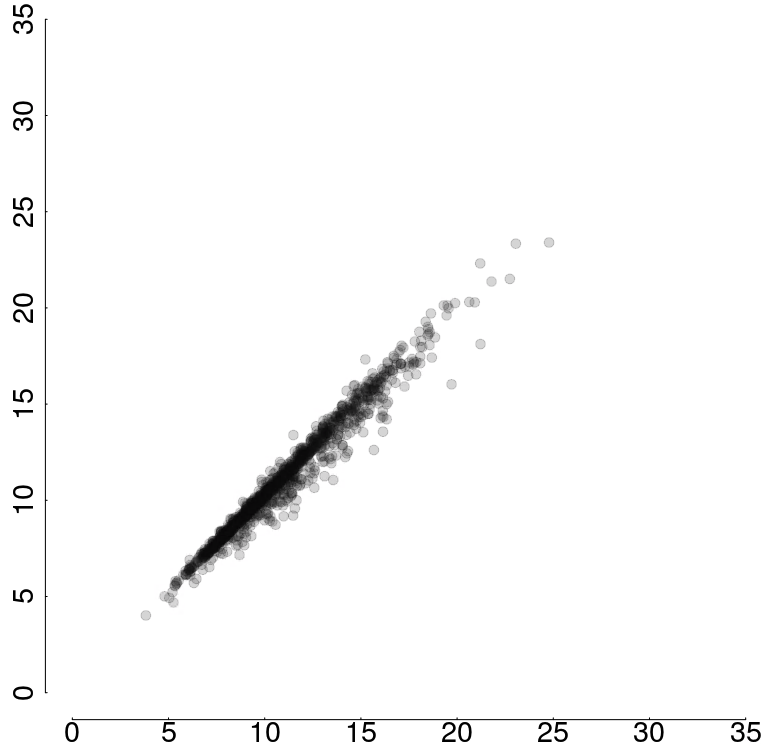


|  | N = 95,915;  I = 1,398 hospitals | N = 88,786;  I = 1,361 hospitals |
| --- | --- | --- |
| Mean  (95% CI) | 10.84  (10.68, 10.99) | 10.98  (10.82, 11.14) |
| Q_0.50_  (Q_0.25_,Q_0.75_) | 10.27  (8.69,12.51) | 10.45  (8.88,12.65) |
| Rank correlation | Kendall coefficient 0.91 (<0.00001) | |

RSER for sensitivity model-2

RSER for sensitivity model-1

S8 Fig. Association between RSERs (%) computed from the models based on the original and sensitivity dataset. Rank correlation coefficient is reported for RSERs based on two models where the first model (X-axis) is based on the main analytical dataset where the second model (Y-axis) utilizes the subset of the main dataset excluding patients who were transferred to short-term, long-term, and critical-care access facilities. Displayed is the rank of common 1,398 hospitals between these two datasets.


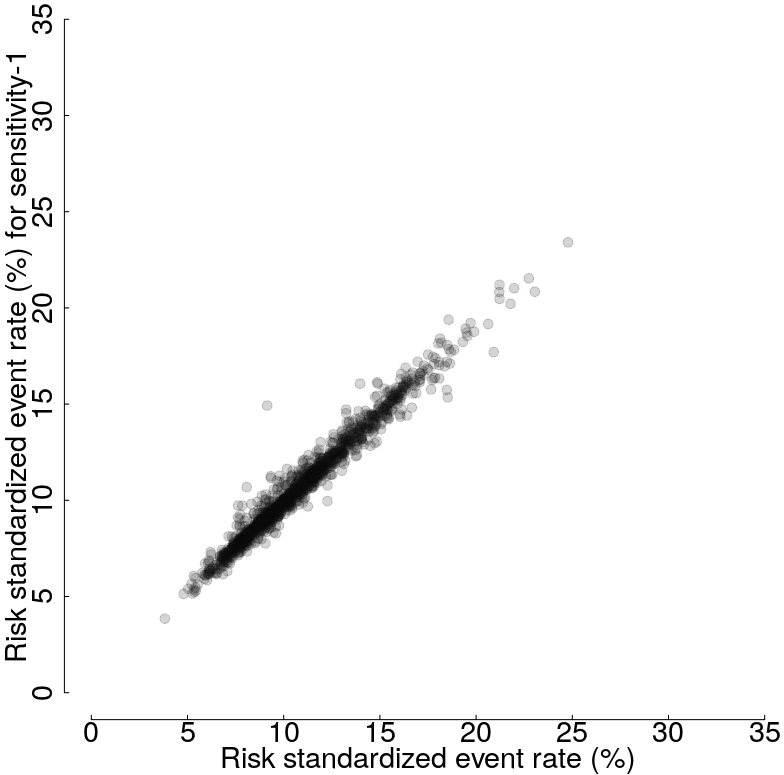


|  | N = 95,915;  I = 1,398 hospitals | N = 103,942;  I = 2,252 hospitals |
| --- | --- | --- |
| Mean  (95% CI) | 10.84  (10.68, 10.99) | 10.68  (10.54, 10.83) |
| Q_0.50_  (Q_0.25_,Q_0.75_) | 10.27  (8.69,12.51) | 10.11  (8.69,12.22) |
| Rank correlation | Kendall coefficient 0.90 (<0.00001) | |

RSER for sensitivity model-2

RSER for sensitivity model-1

S9 Fig. Left panel shows the prevalence of risk factors between early (March 1-May 31, 2020) (X-axis) and late (October 1-Dcemeber 31) (Y-axis) surges based on the main analytical dataset with 43,893 patients admitted across 861 hospitals. Right panel exhibits the counts. Patients with more comorbidity were seen in the early surge comparing to the late surge.


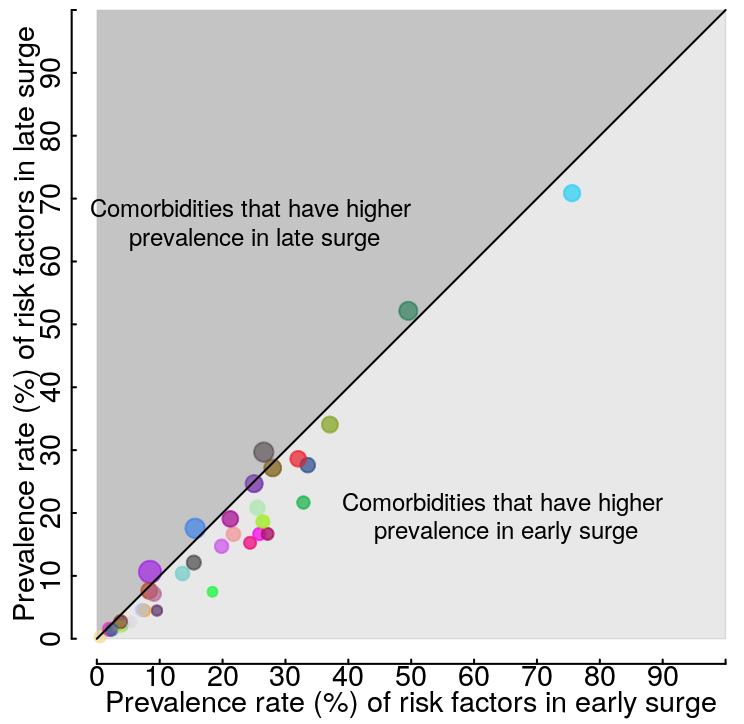

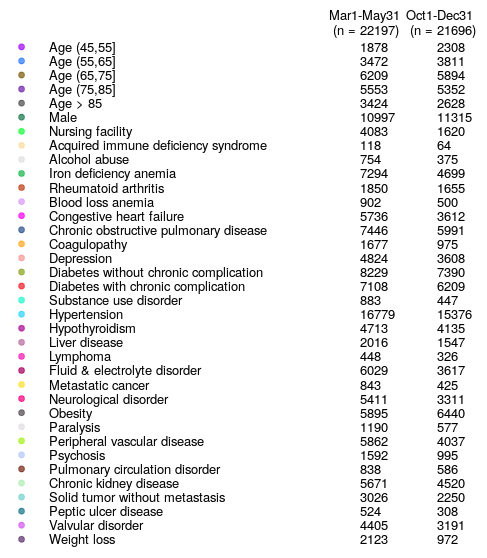


Figure SM10. (A) Bland Altman plot for changes (absolute and relative) in RSERs (%) between early (March 1-May 31, 2020) (X-axis) and late (October 1-Dcemeber 31) (Y-axis) surges based on the main analytical dataset with 43,893 patients admitted across 861 hospitals. (B) RSERs for early (left) and late (right) phase.


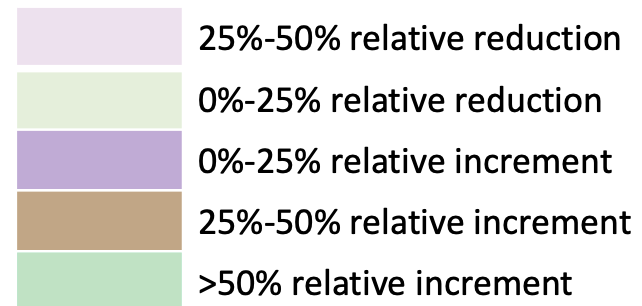

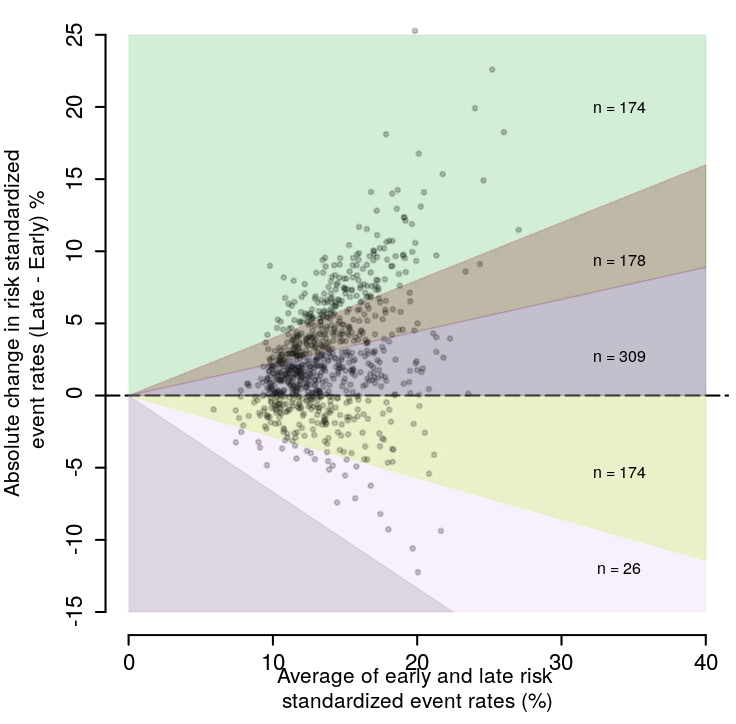


(A)


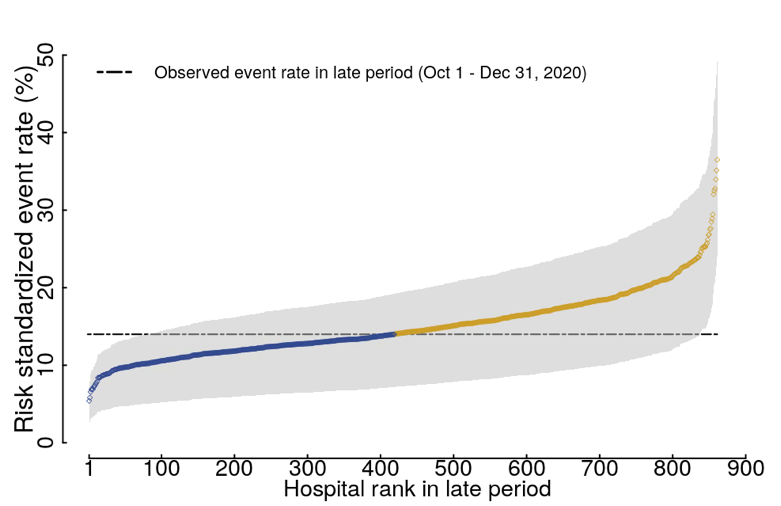

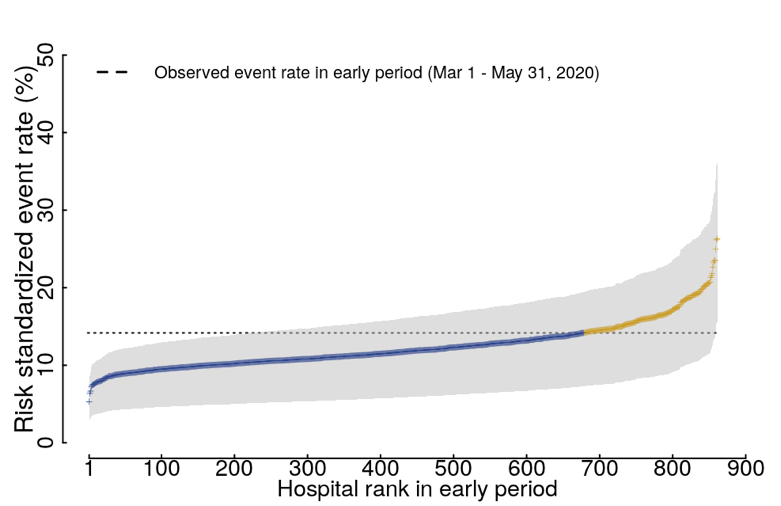


|  | RSER |
| --- | --- |
| Mean (95% CI) | 12.35 (12.16,12.55) |
| Q_0.50_ (IQR) | 11.72 (3.38) |
| Range | 21.03 |

|  | RSER |
| --- | --- |
| Mean (95% CI) | 14.98 (14.70,15.27) |
| Q_0.50_ (IQR) | 14.18 (5.36) |
| Range | 31.08 |

(B)

S1 Table: ICD-10 codes used to define variables.

**Inclusion Criteria for COVID diagnosis**

| Inclusion Criteria | ICD-10-CM codes |
| --- | --- |
| COVID-19 | U071, U072, B9729 |

**Elixhauser Comorbidity Indices**

| Elixhauser Comorbidity Index | ICD-10-CM codes |
| --- | --- |
| Acquired immunodeficiency Syndrome | B20 |
| Alcohol Use Disorder | F1010, F1011, F10120, F10121, F10129, F1014, F10150, F10151, F10159, F10180, F10181, F10182, F10188, F1019, F1020, F1021, F10220, F10221, F10229, F10230, F10231, F10232, F10239, F1024, F10250, F10251, F10259, F1026, F1027, F10280, F10281, F10282, F10288, F1029, F10921, F1094, F10950, F10951, F10959, F1096, F1097, F10980, F10981, F10982, F10988, F1099 |
| Iron Deficiency Anemia | D501, D508, D509, D510, D511, D512, D513, D518, D519, D520, D521, D528, D529, D530, D531, D532, D538, D539, D630, D631, D638, D649 |
| Rheumatoid Arthritis | L900, L940, L941, L943, M0500, M05011, M05012, M05019, M05021, M05022, M05029, M05031, M05032, M05039, M05041, M05042, M05049, M05051, M05052, M05059, M05061, M05062, M05069, M05071, M05072, M05079, M0509, M0510, M05111, M05112, M05119, M05121, M05122, M05129, M05131, M05132, M05139, M05141, M05142, M05149, M05151, M05152, M05159, M05161, M05162, M05169, M05171, M05172, M05179, M0519, M0520, M05211, M05212, M05219, M05221, M05222, M05229, M05231, M05232, M05239, M05241, M05242, M05249, M05251, M05252, M05259, M05261, M05262, M05269, M05271, M05272, M05279, M0529, M0530, M05311, M05312, M05319, M05321, M05322, M05329, M05331, M05332, M05339, M05341, M05342, M05349, M05351, M05352, M05359, M05361, M05362, M05369, M05371, M05372, M05379, M0539, M0540, M05411, M05412, M05419, M05421, M05422, M05429, M05431, M05432, M05439, M05441, M05442, M05449, M05451, M05452, M05459, M05461, M05462, M05469, M05471, M05472, M05479, M0549, M0550, M05511, M05512, M05519, M05521, M05522, M05529, M05531, M05532, M05539, M05541, M05542, M05549, M05551, M05552, M05559, M05561, M05562, M05569, M05571, M05572, M05579, M0559, M0560, M05611, M05612, M05619, M05621, M05622, M05629, M05631, M05632, M05639, M05641, M05642, M05649, M05651, M05652, M05659, M05661, M05662, M05669, M05671, M05672, M05679, M0569, M0570, M05711, M05712, M05719, M05721, M05722, M05729, M05731, M05732, M05739, M05741, M05742, M05749, M05751, M05752, M05759, M05761, M05762, M05769, M05771, M05772, M05779, M0579, M0580, M05811, M05812, M05819, M05821, M05822, M05829, M05831, M05832, M05839, M05841, M05842, M05849, M05851, M05852, M05859, M05861, M05862, M05869, M05871, M05872, M05879, M0589, M059, M0600, M06011, M06012, M06019, M06021, M06022, M06029, M06031, M06032, M06039, M06041, M06042, M06049, M06051, M06052, M06059, M06061, M06062, M06069, M06071, M06072, M06079, M0608, M0609, M061, M0620, M06211, M06212, M06219, M06221, M06222, M06229, M06231, M06232, M06239, M06241, M06242, M06249, M06251, M06252, M06259, M06261, M06262, M06269, M06271, M06272, M06279, M0628, M0629, M0630, M06311, M06312, M06319, M06321, M06322, M06329, M06331, M06332, M06339, M06341, M06342, M06349, M06351, M06352, M06359, M06361, M06362, M06369, M06371, M06372, M06379, M0638, M0639, M064, M0680, M06811, M06812, M06819, M06821, M06822, M06829, M06831, M06832, M06839, M06841, M06842, M06849, M06851, M06852, M06859, M06861, M06862, M06869, M06871, M06872, M06879, M0688, M0689, M069, M0800, M08011, M08012, M08019, M08021, M08022, M08029, M08031, M08032, M08039, M08041, M08042, M08049, M08051, M08052, M08059, M08061, M08062, M08069, M08071, M08072, M08079, M0808, M0809, M081, M0820, M08211, M08212, M08219, M08221, M08222, M08229, M08231, M08232, M08239, M08241, M08242, M08249, M08251, M08252, M08259, M08261, M08262, M08269, M08271, M08272, M08279, M0828, M0829, M083, M0840, M08411, M08412, M08419, M08421, M08422, M08429, M08431, M08432, M08439, M08441, M08442, M08449, M08451, M08452, M08459, M08461, M08462, M08469, M08471, M08472, M08479, M0848, M0880, M08811, M08812, M08819, M08821, M08822, M08829, M08831, M08832, M08839, M08841, M08842, M08849, M08851, M08852, M08859, M08861, M08862, M08869, M08871, M08872, M08879, M0888, M0889, M0890, M08911, M08912, M08919, M08921, M08922, M08929, M08931, M08932, M08939, M08941, M08942, M08949, M08951, M08952, M08959, M08961, M08962, M08969, M08971, M08972, M08979, M0898, M0899, M1200, M12011, M12012, M12019, M12021, M12022, M12029, M12031, M12032, M12039, M12041, M12042, M12049, M12051, M12052, M12059, M12061, M12062, M12069, M12071, M12072, M12079, M1208, M1209, M320, M3210, M3211, M3212, M3213, M3214, M3215, M3219, M328, M329, M3300, M3301, M3302, M3303, M3309, M3310, M3311, M3312, M3313, M3319, M3320, M3321, M3322, M3329, M3390, M3391, M3392, M3393, M3399, M340, M341, M342, M3481, M3482, M3483, M3489, M349, M3500, M3501, M3502, M3503, M3504, M3509, M351, M353, M355, M358, M359, M360, M368, M450, M451, M452, M453, M454, M455, M456, M457, M458, M459, M4600, M4601, M4602, M4603, M4604, M4605, M4606, M4607, M4608, M4609, M461, M4650, M4651, M4652, M4653, M4654, M4655, M4656, M4657, M4658, M4659, M4680, M4681, M4682, M4683, M4684, M4685, M4686, M4687, M4688, M4689, M4690, M4691, M4692, M4693, M4694, M4695, M4696, M4697, M4698, M4699, M488X1, M488X2, M488X3, M488X4, M488X5, M488X6, M488X7, M488X8, M488X9, M4980, M4981, M4982, M4983, M4984, M4985, M4986, M4987, M4988, M4989 |
| Blood Loss Anemia | D500, O9081, O99011, O99012, O99013, O99019, O9902, O9903 |
| Congestive Heart Failure | I0981, I110, I130, I132, I501, I5020, I5021, I5022, I5023, I5030, I5031, I5032, I5033, I5040, I5041, I5042, I5043, I50810, I50811, I50812, I50813, I50814, I5082, I5083, I5084, I5089, I509 |
| Chronic Obstructive Pulmonary Disease | J40, J410, J411, J418, J42, J430, J431, J432, J438, J439, J440, J441, J449, J4520, J4521, J4522, J4530, J4531, J4532, J4540, J4541, J4542, J4550, J4551, J4552, J45901, J45902, J45909, J45990, J45991, J45998, J470, J471, J479, J60, J61, J620, J628, J630, J631, J632, J633, J634, J635, J636, J64, J660, J661, J662, J668, J670, J671, J672, J673, J674, J675, J676, J677, J678, J679, J684 |
| Coagulopathy | D65, D66, D67, D680, D681, D682, D68311, D68312, D68318, D6832, D684, D688, D689, D691, D693, D6941, D6942, D6949, D6951, D6959, D696, D7582, O99111, O99112, O99113, O99119, O9912, O9913 |
| Depression | F320, F321, F322, F323, F328, F3281, F3289, F329, F330, F331, F332, F333, F338, F339, F341, F4321 |
| Diabetes without Chronic Complications | E0800, E0801, E0810, E0811, E089, E0900, E0901, E0910, E0911, E099, E1010, E1011, E109, E1100, E1101, E1110, E1111, E119, E1300, E1301, E1310, E1311, E139, O24011, O24012, O24013, O24019, O2402, O2403, O24111, O24112, O24113, O24119, O2412, O2413, O24311, O24312, O24313, O24319, O2432, O2433, O24811, O24812, O24813, O24819, O2482, O2483, O24911, O24912, O24913, O24919, O2492, O2493 |
| Diabetes with Chronic Complications | E0821, E0822, E0829, E08311, E08319, E08321, E083211, E083212, E083213, E083219, E08329, E083291, E083292, E083293, E083299, E08331, E083311, E083312, E083313, E083319, E08339, E083391, E083392, E083393, E083399, E08341, E083411, E083412, E083413, E083419, E08349, E083491, E083492, E083493, E083499, E08351, E083511, E083512, E083513, E083519, E083521, E083522, E083523, E083529, E083531, E083532, E083533, E083539, E083541, E083542, E083543, E083549, E083551, E083552, E083553, E083559, E08359, E083591, E083592, E083593, E083599, E0836, E0837X1, E0837X2, E0837X3, E0837X9, E0839, E0840, E0841, E0842, E0843, E0844, E0849, E0851, E0852, E0859, E08610, E08618, E08620, E08621, E08622, E08628, E08630, E08638, E08641, E08649, E0865, E0869, E088, E0921, E0922, E0929, E09311, E09319, E09321, E093211, E093212, E093213, E093219, E09329, E093291, E093292, E093293, E093299, E09331, E093311, E093312, E093313, E093319, E09339, E093391, E093392, E093393, E093399, E09341, E093411, E093412, E093413, E093419, E09349, E093491, E093492, E093493, E093499, E09351, E093511, E093512, E093513, E093519, E093521, E093522, E093523, E093529, E093531, E093532, E093533, E093539, E093541, E093542, E093543, E093549, E093551, E093552, E093553, E093559, E09359, E093591, E093592, E093593, E093599, E0936, E0937X1, E0937X2, E0937X3, E0937X9, E0939, E0940, E0941, E0942, E0943, E0944, E0949, E0951, E0952, E0959, E09610, E09618, E09620, E09621, E09622, E09628, E09630, E09638, E09641, E09649, E0965, E0969, E098, E1021, E1022, E1029, E10311, E10319, E10321, E103211, E103212, E103213, E103219, E10329, E103291, E103292, E103293, E103299, E10331, E103311, E103312, E103313, E103319, E10339, E103391, E103392, E103393, E103399, E10341, E103411, E103412, E103413, E103419, E10349, E103491, E103492, E103493, E103499, E10351, E103511, E103512, E103513, E103519, E103521, E103522, E103523, E103529, E103531, E103532, E103533, E103539, E103541, E103542, E103543, E103549, E103551, E103552, E103553, E103559, E10359, E103591, E103592, E103593, E103599, E1036, E1037X1, E1037X2, E1037X3, E1037X9, E1039, E1040, E1041, E1042, E1043, E1044, E1049, E1051, E1052, E1059, E10610, E10618, E10620, E10621, E10622, E10628, E10630, E10638, E10641, E10649, E1065, E1069, E108, E1121, E1122, E1129, E11311, E11319, E11321, E113211, E113212, E113213, E113219, E11329, E113291, E113292, E113293, E113299, E11331, E113311, E113312, E113313, E113319, E11339, E113391, E113392, E113393, E113399, E11341, E113411, E113412, E113413, E113419, E11349, E113491, E113492, E113493, E113499, E11351, E113511, E113512, E113513, E113519, E113521, E113522, E113523, E113529, E113531, E113532, E113533, E113539, E113541, E113542, E113543, E113549, E113551, E113552, E113553, E113559, E11359, E113591, E113592, E113593, E113599, E1136, E1137X1, E1137X2, E1137X3, E1137X9, E1139, E1140, E1141, E1142, E1143, E1144, E1149, E1151, E1152, E1159, E11610, E11618, E11620, E11621, E11622, E11628, E11630, E11638, E11641, E11649, E1165, E1169, E118, E1321, E1322, E1329, E13311, E13319, E13321, E133211, E133212, E133213, E133219, E13329, E133291, E133292, E133293, E133299, E13331, E133311, E133312, E133313, E133319, E13339, E133391, E133392, E133393, E133399, E13341, E133411, E133412, E133413, E133419, E13349, E133491, E133492, E133493, E133499, E13351, E133511, E133512, E133513, E133519, E133521, E133522, E133523, E133529, E133531, E133532, E133533, E133539, E133541, E133542, E133543, E133549, E133551, E133552, E133553, E133559, E13359, E133591, E133592, E133593, E133599, E1336, E1337X1, E1337X2, E1337X3, E1337X9, E1339, E1340, E1341, E1342, E1343, E1344, E1349, E1351, E1352, E1359, E13610, E13618, E13620, E13621, E13622, E13628, E13630, E13638, E13641, E13649, E1365, E1369, E138, P702 |
| Substance Use Disorder | F1110, F1111, F11120, F11121, F11122, F11129, F1114, F11150, F11151, F11159, F11181, F11182, F11188, F1119, F1120, F1121, F11220, F11221, F11222, F11229, F1123, F1124, F11250, F11251, F11259, F11281, F11282, F11288, F1129, F1210, F1211, F12120, F12121, F12122, F12129, F12150, F12151, F12159, F12180, F12188, F1219, F1220, F1221, F12220, F12221, F12222, F12229, F1223, F12250, F12251, F12259, F12280, F12288, F1229, F1310, F1311, F13120, F13121, F13129, F1314, F13150, F13151, F13159, F13180, F13181, F13182, F13188, F1319, F1320, F1321, F13220, F13221, F13229, F13230, F13231, F13232, F13239, F1324, F13250, F13251, F13259, F1326, F1327, F13280, F13281, F13282, F13288, F1329, F1410, F1411, F14120, F14121, F14122, F14129, F1414, F14150, F14151, F14159, F14180, F14181, F14182, F14188, F1419, F1420, F1421, F14220, F14221, F14222, F14229, F1423, F1424, F14250, F14251, F14259, F14280, F14281, F14282, F14288, F1429, F1510, F1511, F15120, F15121, F15122, F15129, F1514, F15150, F15151, F15159, F15180, F15181, F15182, F15188, F1519, F1520, F1521, F15220, F15221, F15222, F15229, F1523, F1524, F15250, F15251, F15259, F15280, F15281, F15282, F15288, F1529, F1610, F1611, F16120, F16121, F16122, F16129, F1614, F16150, F16151, F16159, F16180, F16183, F16188, F1619, F1620, F1621, F16220, F16221, F16229, F1624, F16250, F16251, F16259, F16280, F16283, F16288, F1629, F1810, F1811, F18120, F18121, F18129, F1814, F18150, F18151, F18159, F1817, F18180, F18188, F1819, F1820, F1821, F18220, F18221, F18229, F1824, F18250, F18251, F18259, F1827, F18280, F18288, F1829, F1910, F1911, F19120, F19121, F19122, F19129, F1914, F19150, F19151, F19159, F1916, F1917, F19180, F19181, F19182, F19188, F1919, F1920, F1921, F19220, F19221, F19222, F19229, F19230, F19231, F19232, F19239, F1924, F19250, F19251, F19259, F1926, F1927, F19280, F19281, F19282, F19288, F1929, F550, F551, F552, F553, F554, F558, O99320, O99321, O99322, O99323, O99324, O99325 |
| Hypertension | I10, I110, I119, I120, I129, I130, I1310, I1311, I132, I150, I151, I152, I158, I159, I160, I161, I169, I674, O10011, O10012, O10013, O10019, O1002, O1003, O10111, O10112, O10113, O10119, O1012, O1013, O10211, O10212, O10213, O10219, O1022, O1023, O10311, O10312, O10313, O10319, O1032, O1033, O10411, O10412, O10413, O10419, O1042, O1043, O10911, O10912, O10913, O10919, O1092, O1093, O111, O112, O113, O114, O115, O119, O161, O162, O163, O164, O165, O169 |
| Hypothyroidism | E000, E001, E002, E009, E018, E02, E030, E031, E032, E033, E038, E039, E890 |
| Liver Disease | B180, B181, B182, I8500, I8501, I8510, I8511, K700, K702, K7030, K7031, K7040, K7041, K709, K7210, K7211, K7290, K7291, K730, K731, K732, K738, K739, K740, K741, K742, K743, K744, K745, K7460, K7469, K754, K7581, K760, K766, K7689, K769, Z944 |
| Lymphoma | C8100, C8101, C8102, C8103, C8104, C8105, C8106, C8107, C8108, C8109, C8110, C8111, C8112, C8113, C8114, C8115, C8116, C8117, C8118, C8119, C8120, C8121, C8122, C8123, C8124, C8125, C8126, C8127, C8128, C8129, C8130, C8131, C8132, C8133, C8134, C8135, C8136, C8137, C8138, C8139, C8140, C8141, C8142, C8143, C8144, C8145, C8146, C8147, C8148, C8149, C8170, C8171, C8172, C8173, C8174, C8175, C8176, C8177, C8178, C8179, C8190, C8191, C8192, C8193, C8194, C8195, C8196, C8197, C8198, C8199, C8200, C8201, C8202, C8203, C8204, C8205, C8206, C8207, C8208, C8209, C8210, C8211, C8212, C8213, C8214, C8215, C8216, C8217, C8218, C8219, C8220, C8221, C8222, C8223, C8224, C8225, C8226, C8227, C8228, C8229, C8230, C8231, C8232, C8233, C8234, C8235, C8236, C8237, C8238, C8239, C8240, C8241, C8242, C8243, C8244, C8245, C8246, C8247, C8248, C8249, C8250, C8251, C8252, C8253, C8254, C8255, C8256, C8257, C8258, C8259, C8260, C8261, C8262, C8263, C8264, C8265, C8266, C8267, C8268, C8269, C8280, C8281, C8282, C8283, C8284, C8285, C8286, C8287, C8288, C8289, C8290, C8291, C8292, C8293, C8294, C8295, C8296, C8297, C8298, C8299, C8300, C8301, C8302, C8303, C8304, C8305, C8306, C8307, C8308, C8309, C8310, C8311, C8312, C8313, C8314, C8315, C8316, C8317, C8318, C8319, C8330, C8331, C8332, C8333, C8334, C8335, C8336, C8337, C8338, C8339, C8350, C8351, C8352, C8353, C8354, C8355, C8356, C8357, C8358, C8359, C8370, C8371, C8372, C8373, C8374, C8375, C8376, C8377, C8378, C8379, C8380, C8381, C8382, C8383, C8384, C8385, C8386, C8387, C8388, C8389, C8390, C8391, C8392, C8393, C8394, C8395, C8396, C8397, C8398, C8399, C8400, C8401, C8402, C8403, C8404, C8405, C8406, C8407, C8408, C8409, C8410, C8411, C8412, C8413, C8414, C8415, C8416, C8417, C8418, C8419, C8440, C8441, C8442, C8443, C8444, C8445, C8446, C8447, C8448, C8449, C8460, C8461, C8462, C8463, C8464, C8465, C8466, C8467, C8468, C8469, C8470, C8471, C8472, C8473, C8474, C8475, C8476, C8477, C8478, C8479, C8490, C8491, C8492, C8493, C8494, C8495, C8496, C8497, C8498, C8499, C84A0, C84A1, C84A2, C84A3, C84A4, C84A5, C84A6, C84A7, C84A8, C84A9, C84Z0, C84Z1, C84Z2, C84Z3, C84Z4, C84Z5, C84Z6, C84Z7, C84Z8, C84Z9, C8510, C8511, C8512, C8513, C8514, C8515, C8516, C8517, C8518, C8519, C8520, C8521, C8522, C8523, C8524, C8525, C8526, C8527, C8528, C8529, C8580, C8581, C8582, C8583, C8584, C8585, C8586, C8587, C8588, C8589, C8590, C8591, C8592, C8593, C8594, C8595, C8596, C8597, C8598, C8599, C860, C861, C862, C863, C864, C865, C866, C880, C882, C883, C884, C888, C889, C9000, C9001, C9002, C9010, C9011, C9012, C9020, C9021, C9022, C9030, C9031, C9032, C960, C962, C9620, C9621, C9622, C9629, C964, C969, C96A, C96Z, D47Z9 |
| Fluid and Electrolyte Disorder | E860, E861, E869, E870, E871, E872, E873, E874, E875, E876, E8770, E8771, E8779, E878 |
| Metastatic Cancer | C770, C771, C772, C773, C774, C775, C778, C779, C7800, C7801, C7802, C781, C782, C7830, C7839, C784, C785, C786, C787, C7880, C7889, C7900, C7901, C7902, C7910, C7911, C7919, C792, C7931, C7932, C7940, C7949, C7951, C7952, C7960, C7961, C7962, C7970, C7971, C7972, C7981, C7982, C7989, C799, C7B00, C7B01, C7B02, C7B03, C7B04, C7B09, C7B1, C7B8, C800, C801, R180 |
| Neurological Disorder | E7500, E7501, E7502, E7509, E7510, E7511, E7519, E7523, E7525, E7526, E7529, E754, F842, G10, G110, G111, G112, G113, G114, G118, G119, G120, G121, G1220, G1221, G1222, G1223, G1224, G1225, G1229, G128, G129, G132, G138, G20, G214, G2401, G2402, G2409, G242, G248, G254, G255, G2581, G300, G301, G308, G309, G3101, G3109, G311, G312, G3181, G3182, G3183, G3184, G3185, G3189, G319, G3281, G35, G361, G368, G369, G370, G371, G372, G373, G374, G375, G378, G379, G40001, G40009, G40011, G40019, G40101, G40109, G40111, G40119, G40201, G40209, G40211, G40219, G40301, G40309, G40311, G40319, G40401, G40409, G40411, G40419, G40501, G40509, G40801, G40802, G40803, G40804, G40811, G40812, G40813, G40814, G40821, G40822, G40823, G40824, G4089, G40901, G40909, G40911, G40919, G40A01, G40A09, G40A11, G40A19, G40B01, G40B09, G40B11, G40B19, G47411, G47419, G47421, G47429, G803, G890, G910, G911, G912, G913, G914, G918, G919, G937, G9389, G939, G94, O99350, O99351, O99352, O99353, O99354, O99355, P9160, P9161, P9162, P9163, R410, R4182, R4701, R5600, R5601, R561, R569 |
| Obesity | E6601, E6609, E661, E662, E668, E669, O99210, O99211, O99212, O99213, O99214, O99215, R939, Z6830, Z6831, Z6832, Z6833, Z6834, Z6835, Z6836, Z6837, Z6838, Z6839, Z6841, Z6842, Z6843, Z6844, Z6845, Z6854 |
| Paralysis | G041, G800, G801, G802, G804, G808, G809, G8100, G8101, G8102, G8103, G8104, G8110, G8111, G8112, G8113, G8114, G8190, G8191, G8192, G8193, G8194, G8220, G8221, G8222, G8250, G8251, G8252, G8253, G8254, G830, G8310, G8311, G8312, G8313, G8314, G8320, G8321, G8322, G8323, G8324, G8330, G8331, G8332, G8333, G8334, G834, G835, G8381, G8382, G8383, G8384, G8389, G839, I69031, I69032, I69033, I69034, I69039, I69041, I69042, I69043, I69044, I69049, I69051, I69052, I69053, I69054, I69059, I69061, I69062, I69063, I69064, I69065, I69069, I69131, I69132, I69133, I69134, I69139, I69141, I69142, I69143, I69144, I69149, I69151, I69152, I69153, I69154, I69159, I69161, I69162, I69163, I69164, I69165, I69169, I69231, I69232, I69233, I69234, I69239, I69241, I69242, I69243, I69244, I69249, I69251, I69252, I69253, I69254, I69259, I69261, I69262, I69263, I69264, I69265, I69269, I69331, I69332, I69333, I69334, I69339, I69341, I69342, I69343, I69344, I69349, I69351, I69352, I69353, I69354, I69359, I69361, I69362, I69363, I69364, I69365, I69369, I69831, I69832, I69833, I69834, I69839, I69841, I69842, I69843, I69844, I69849, I69851, I69852, I69853, I69854, I69859, I69861, I69862, I69863, I69864, I69865, I69869, I69931, I69932, I69933, I69934, I69939, I69941, I69942, I69943, I69944, I69949, I69951, I69952, I69953, I69954, I69959, I69961, I69962, I69963, I69964, I69965, I69969, R532 |
| Peripheral Vascular Disease | I700, I701, I70201, I70202, I70203, I70208, I70209, I70211, I70212, I70213, I70218, I70219, I70221, I70222, I70223, I70228, I70229, I70231, I70232, I70233, I70234, I70235, I70238, I70239, I70241, I70242, I70243, I70244, I70245, I70248, I70249, I7025, I70261, I70262, I70263, I70268, I70269, I70291, I70292, I70293, I70298, I70299, I70301, I70302, I70303, I70308, I70309, I70311, I70312, I70313, I70318, I70319, I70321, I70322, I70323, I70328, I70329, I70331, I70332, I70333, I70334, I70335, I70338, I70339, I70341, I70342, I70343, I70344, I70345, I70348, I70349, I7035, I70361, I70362, I70363, I70368, I70369, I70391, I70392, I70393, I70398, I70399, I70401, I70402, I70403, I70408, I70409, I70411, I70412, I70413, I70418, I70419, I70421, I70422, I70423, I70428, I70429, I70431, I70432, I70433, I70434, I70435, I70438, I70439, I70441, I70442, I70443, I70444, I70445, I70448, I70449, I7045, I70461, I70462, I70463, I70468, I70469, I70491, I70492, I70493, I70498, I70499, I70501, I70502, I70503, I70508, I70509, I70511, I70512, I70513, I70518, I70519, I70521, I70522, I70523, I70528, I70529, I70531, I70532, I70533, I70534, I70535, I70538, I70539, I70541, I70542, I70543, I70544, I70545, I70548, I70549, I7055, I70561, I70562, I70563, I70568, I70569, I70591, I70592, I70593, I70598, I70599, I70601, I70602, I70603, I70608, I70609, I70611, I70612, I70613, I70618, I70619, I70621, I70622, I70623, I70628, I70629, I70631, I70632, I70633, I70634, I70635, I70638, I70639, I70641, I70642, I70643, I70644, I70645, I70648, I70649, I7065, I70661, I70662, I70663, I70668, I70669, I70691, I70692, I70693, I70698, I70699, I70701, I70702, I70703, I70708, I70709, I70711, I70712, I70713, I70718, I70719, I70721, I70722, I70723, I70728, I70729, I70731, I70732, I70733, I70734, I70735, I70738, I70739, I70741, I70742, I70743, I70744, I70745, I70748, I70749, I7075, I70761, I70762, I70763, I70768, I70769, I70791, I70792, I70793, I70798, I70799, I708, I7090, I7091, I7092, I7100, I7101, I7102, I7103, I711, I712, I713, I714, I715, I716, I718, I719, I720, I721, I722, I723, I724, I725, I726, I728, I729, I731, I7381, I7389, I739, I742, I743, I744, I76, I771, I7770, I7771, I7772, I7773, I7774, I7775, I7776, I7777, I7779, I790, I791, I798, K551, K558, K559, Z95820, Z95828 |
| Psychosis | F200, F201, F202, F203, F205, F2081, F2089, F209, F22, F23, F24, F250, F251, F258, F259, F28, F29, F3010, F3011, F3012, F3013, F302, F303, F304, F308, F309, F310, F3110, F3111, F3112, F3113, F312, F3130, F3131, F3132, F314, F315, F3160, F3161, F3162, F3163, F3164, F3170, F3171, F3172, F3173, F3174, F3175, F3176, F3177, F3178, F3181, F3189, F319, F324, F325, F3340, F3341, F3342, F348, F3481, F3489, F349, F39, F4489, F843 |
| Pulmonary Circulation | I2601, I2602, I2609, I2690, I2692, I2699, I270, I271, I2781, I2782, I2783, I2789, I279, I289, T800XXA, T82817A, T82818A |
| Chronic Kidney Disease | I120, I1311, I132, N183, N184, N185, N186, N189, N19, Z4901, Z4902, Z4931, Z4932, Z9115, Z940, Z992 |
| Solid Tumor without Metastasis | C000, C001, C002, C003, C004, C005, C006, C008, C009, C01, C020, C021, C022, C023, C024, C028, C029, C030, C031, C039, C040, C041, C048, C049, C050, C051, C052, C058, C059, C060, C061, C062, C0680, C0689, C069, C07, C080, C081, C089, C090, C091, C098, C099, C100, C101, C102, C103, C104, C108, C109, C110, C111, C112, C113, C118, C119, C12, C130, C131, C132, C138, C139, C140, C142, C148, C153, C154, C155, C158, C159, C160, C161, C162, C163, C164, C165, C166, C168, C169, C170, C171, C172, C173, C178, C179, C180, C181, C182, C183, C184, C185, C186, C187, C188, C189, C19, C20, C210, C211, C212, C218, C220, C221, C222, C223, C224, C227, C228, C229, C23, C240, C241, C248, C249, C250, C251, C252, C253, C254, C257, C258, C259, C260, C261, C269, C300, C301, C310, C311, C312, C313, C318, C319, C320, C321, C322, C323, C328, C329, C33, C3400, C3401, C3402, C3410, C3411, C3412, C342, C3430, C3431, C3432, C3480, C3481, C3482, C3490, C3491, C3492, C37, C380, C381, C382, C383, C384, C388, C390, C399, C4000, C4001, C4002, C4010, C4011, C4012, C4020, C4021, C4022, C4030, C4031, C4032, C4080, C4081, C4082, C4090, C4091, C4092, C410, C411, C412, C413, C414, C419, C430, C4310, C4311, C43111, C43112, C4312, C43121, C43122, C4320, C4321, C4322, C4330, C4331, C4339, C434, C4351, C4352, C4359, C4360, C4361, C4362, C4370, C4371, C4372, C438, C439, C450, C451, C452, C457, C470, C4710, C4711, C4712, C4720, C4721, C4722, C473, C474, C475, C476, C478, C479, C480, C481, C482, C488, C490, C4910, C4911, C4912, C4920, C4921, C4922, C493, C494, C495, C496, C498, C499, C49A0, C49A1, C49A2, C49A3, C49A4, C49A5, C49A9, C4A0, C4A10, C4A11, C4A111, C4A112, C4A12, C4A121, C4A122, C4A20, C4A21, C4A22, C4A30, C4A31, C4A39, C4A4, C4A51, C4A52, C4A59, C4A60, C4A61, C4A62, C4A70, C4A71, C4A72, C4A8, C4A9, C50011, C50012, C50019, C50021, C50022, C50029, C50111, C50112, C50119, C50121, C50122, C50129, C50211, C50212, C50219, C50221, C50222, C50229, C50311, C50312, C50319, C50321, C50322, C50329, C50411, C50412, C50419, C50421, C50422, C50429, C50511, C50512, C50519, C50521, C50522, C50529, C50611, C50612, C50619, C50621, C50622, C50629, C50811, C50812, C50819, C50821, C50822, C50829, C50911, C50912, C50919, C50921, C50922, C50929, C510, C511, C512, C518, C519, C52, C530, C531, C538, C539, C540, C541, C542, C543, C548, C549, C55, C561, C562, C569, C5700, C5701, C5702, C5710, C5711, C5712, C5720, C5721, C5722, C573, C574, C577, C578, C579, C58, C600, C601, C602, C608, C609, C61, C6200, C6201, C6202, C6210, C6211, C6212, C6290, C6291, C6292, C6300, C6301, C6302, C6310, C6311, C6312, C632, C637, C638, C639, C641, C642, C649, C651, C652, C659, C661, C662, C669, C670, C671, C672, C673, C674, C675, C676, C677, C678, C679, C680, C681, C688, C689, C6900, C6901, C6902, C6910, C6911, C6912, C6920, C6921, C6922, C6930, C6931, C6932, C6940, C6941, C6942, C6950, C6951, C6952, C6960, C6961, C6962, C6980, C6981, C6982, C6990, C6991, C6992, C700, C701, C709, C710, C711, C712, C713, C714, C715, C716, C717, C718, C719, C720, C721, C7220, C7221, C7222, C7230, C7231, C7232, C7240, C7241, C7242, C7250, C7259, C729, C73, C7400, C7401, C7402, C7410, C7411, C7412, C7490, C7491, C7492, C750, C751, C752, C753, C754, C755, C758, C759, C760, C761, C762, C763, C7640, C7641, C7642, C7650, C7651, C7652, C768, C7A00, C7A010, C7A011, C7A012, C7A019, C7A020, C7A021, C7A022, C7A023, C7A024, C7A025, C7A026, C7A029, C7A090, C7A091, C7A092, C7A093, C7A094, C7A095, C7A096, C7A098, D030, D0310, D0311, D03111, D03112, D0312, D03121, D03122, D0320, D0321, D0322, D0330, D0339, D034, D0351, D0352, D0359, D0360, D0361, D0362, D0370, D0371, D0372, D038, D039, E3121, E3122, E3123 |
| Peptic Ulcer | K254, K255, K256, K257, K259, K264, K265, K266, K267, K269, K274, K275, K276, K277, K279, K284, K285, K286, K287, K289 |
| Valvular Disorder | A5203, I050, I051, I052, I058, I059, I060, I061, I062, I068, I069, I070, I071, I072, I078, I079, I080, I081, I082, I083, I088, I089, I091, I0989, I340, I341, I342, I348, I349, I350, I351, I352, I358, I359, I360, I361, I362, I368, I369, I370, I371, I372, I378, I379, I38, I39, Q230, Q231, Q232, Q233, Z952, Z953, Z954 |
| Weight Loss | E40, E41, E42, E43, E440, E441, E45, E46, E640, R634, R636 |

S2 Table. Baseline characteristics for 95,919 COVID hospitalized patients. Reported are the mean and standard deviation for numeric variables, and prevalence counts and percentage (%) for categorical variables.

| Variables | Summary measures |
| --- | --- |
| N | 95915 |
| Age (mean (SD)) | 68.99 (15.62) |
| Age bucket (%) |  |
| 18 ≤ age ≤ 45 | 8260 ( 8.6) |
| 45 < age ≤ 55 | 9244 ( 9.6) |
| 55 < age ≤ 65 | 16162 (16.9) |
| 65 < age ≤ 75 | 26243 (27.4) |
| 75 < age ≤ 85 | 23202 (24.2) |
| 85 < age | 12804 (13.3) |
| Gender = Male (%) | 47583 (49.6) |
| Transferred from nursing facility = Yes (%) | 12003 (12.5) |
| Elixhauser comorbidities, Count (%) |  |
| Acquired immune deficiency syndrome | 404 ( 0.4) |
| Alcohol abuse | 2688 ( 2.8) |
| Iron deficiency anemia | 26211 (27.3) |
| Rheumatoid arthritis | 7690 ( 8.0) |
| Blood loss anemia | 3126 ( 3.3) |
| Congestive heart failure | 20033 (20.9) |
| Chronic obstructive pulmonary disease | 29026 (30.3) |
| Coagulopathy | 5781 ( 6.0) |
| Depression | 18554 (19.3) |
| Diabetes without chronic complication | 33818 (35.3) |
| Diabetes with chronic complication | 28950 (30.2) |
| Substance use disorder | 3069 ( 3.2) |
| Hypertension | 70231 (73.2) |
| Hypothyroidism | 19353 (20.2) |
| Liver disease | 8095 ( 8.4) |
| Lymphoma | 1622 ( 1.7) |
| Fluid & electrolyte disorder | 21266 (22.2) |
| Metastatic cancer | 2927 ( 3.1) |
| Neurological disorder | 18838 (19.6) |
| Obesity | 27057 (28.2) |
| Paralysis | 3839 ( 4.0) |
| Peripheral vascular disease | 21104 (22.0) |
| Psychosis | 5535 ( 5.8) |
| Pulmonary circulation disorder | 3079 ( 3.2) |
| Chronic kidney disease | 21923 (22.9) |
| Solid tumor without metastasis | 11529 (12.0) |
| Peptic ulcer disease | 1951 ( 2.0) |
| Valvular disorder | 16581 (17.3) |
| Weight loss | 6738 ( 7.0) |
| Days from March 1,2020 (Count, %) |  |
| 0 < Count ≤ 30 | 3514 ( 3.7) |
| 30 < Count ≤ 60 | 10241 (10.7) |
| 60 < Count ≤ 90 | 10547 (11.0) |
| 90 < Count ≤ 120 | 10376 (10.8) |
| 120 < Count ≤ 150 | 13448 (14.0) |
| 150 < Count ≤ 180 | 12197 (12.7) |
| 180 < Count ≤ 210 | 9259 ( 9.7) |
| 210 < Count ≤ 240 | 5147 ( 5.4) |
| 240 < Count ≤ 270 | 8545 ( 8.9) |
| 270 < Count | 12641 (13.2) |
| Logarithm of patient volume (mean (SD)) | 9.60 (0.80) |
| Composite observed mortality (Count, %) | 10688 (11.1) |

S3 Table. Sensitivity analysis: 12 months of enrollment filter in 2019

| ***Variables*** | ***Estimates*** | ***P Value*** | ***90% CI*** | ***95% CI*** |
| --- | --- | --- | --- | --- |
| (Intercept) | -2.155 | **<0.001** | (-2.267, -2.038) | (-2.291, -2.014) |
| RN | -0.013 | 0.161 | (-0.028, 0.002) | (-0.031, 0.005) |
| Hospitalists | -0.005 | 0.555 | (-0.023, 0.006) | (-0.026, 0.009) |
| Intensivists | -0.006 | 0.483 | (-0.020, 0.008) | (-0.022, 0.011) |
| Emergency  physicians | -0.020 | **0.020** | (-0.034, -0.006) | (-0.036, -0.004) |
| # hospital beds | 0.034 | **0.015** | (0.012, 0.059) | (0.006, 0.062) |
| # medical/surgical  ICU beds | -0.000 | 0.979 | (-0.022, 0.021) | (-0.027, 0.026) |
| # airborne infection  isolation rooms | -0.008 | 0.430 | (-0.024, 0.007) | (-0.027, 0.009) |
| Not‐for‐profit  (reference) |  |  |  |  |
| For-profit | -0.040 | 0.072 | (-0.078, -0.001) | (-0.088, 0.008) |
| Local public | -0.012 | 0.620 | (-0.059, 0.034) | (-0.067, 0.045) |
| One or more  ACGME programs | 0.057 | **0.001** | (0.028, 0.088) | (0.022, 0.094) |
| Magnet | -0.032 | 0.125 | (-0.067, 0.001) | (-0.072, 0.006) |
| Occupancy  rate | 0.032 | **0.001** | (0.017, 0.050) | (0.014, 0.053) |
| Medicare share | 0.009 | 0.402 | (-0.008, 0.026) | (-0.012, 0.030) |
| Medicaid share | 0.010 | 0.361 | (-0.007, 0.029) | (-0.011, 0.032) |
| Urban county | 0.085 | **0.001** | (0.041, 0.132) | (0.033, 0.139) |
| HHI | 0.007 | 0.393 | (-0.007, 0.022) | (-0.010, 0.025) |
| SVI Theme 1 | -0.002 | 0.881 | (-0.023, 0.019) | (-0.028, 0.023) |
| SVI Theme 2 | 0.008 | 0.423 | (-0.009, 0.025) | (-0.012, 0.029) |
| SVI Theme 3 | -0.011 | 0.248 | (-0.027, 0.005) | (-0.031, 0.009) |
| SVI Theme 4 | 0.017 | 0.071 | (0.000, 0.035) | (-0.004, 0.037) |
| COVID case rate | 0.031 | **0.003** | (0.013, 0.050) | (0.011, 0.055) |

**S4 Table.** **Sensitivity analysis: the counts of UHG enrolled-hospitalized patients represent the corresponding size of hospitals**

| ***Variables*** | ***Estimates*** | ***P Value*** | ***90% CI*** | ***95% CI*** |
| --- | --- | --- | --- | --- |
| (Intercept) | -2.148 | **<0.001** | (-2.264, -2.036) | (-2.287, -2.007) |
| RN | -0.017 | 0.068 | (-0.032, -0.002) | (-0.035, 0.000) |
| Hospitalists | -0.006 | 0.516 | (-0.024, 0.005) | (-0.028, 0.008) |
| Intensivists | -0.005 | 0.493 | (-0.018, 0.008) | (-0.02, 0.011) |
| Emergency  physicians | -0.014 | 0.112 | (-0.030, 0.000) | (-0.034, 0.002) |
| # hospital beds | 0.019 | 0.197 | (-0.007, 0.045) | (-0.013, 0.048) |
| # medical/surgical  ICU beds | 0.000 | 0.997 | (-0.022, 0.022) | (-0.026, 0.027) |
| # airborne infection  isolation rooms | -0.005 | 0.607 | (-0.022, 0.011) | (-0.025, 0.014) |
| Not‐for‐profit  (reference) |  |  |  |  |
| For-profit | -0.041 | 0.068 | (-0.079, -0.004) | (-0.090, 0.002) |
| Local public | -0.007 | 0.786 | (-0.052, 0.041) | (-0.060, 0.048) |
| One or more  ACGME programs | 0.056 | **0.002** | (0.024, 0.086) | (0.018, 0.091) |
| Magnet | -0.031 | 0.162 | (-0.065, 0.005) | (-0.071, 0.011) |
| Occupancy  rate | 0.019 | 0.051 | (0.002, 0.037) | (-0.002, 0.040) |
| Medicare share | 0.013 | 0.217 | (-0.005, 0.030) | (-0.008, 0.035) |
| Medicaid share | 0.012 | 0.275 | (-0.007, 0.030) | (-0.011, 0.035) |
| Urban county | 0.079 | **0.004** | (0.031, 0.128) | (0.024, 0.135) |
| HHI | 0.006 | 0.474 | (-0.009, 0.022) | (-0.011, 0.026) |
| SVI Theme 1 | -0.002 | 0.888 | (-0.023, 0.019) | (-0.027, 0.024) |
| SVI Theme 2 | 0.010 | 0.340 | (-0.007, 0.028) | (-0.012, 0.033) |
| SVI Theme 3 | -0.010 | 0.301 | (-0.026, 0.006) | (-0.030, 0.011) |
| SVI Theme 4 | 0.013 | 0.180 | (-0.003, 0.031) | (-0.007, 0.035) |
| COVID case rate | 0.036 | **0.001** | (0.016, 0.056) | (0.013, 0.059) |
